# The Function of Photocatalytic Performance and Carrier Separation Efficiency Tuned by Doping Content in Homogeneous Photocatalysts

**DOI:** 10.1002/advs.202501026

**Published:** 2025-04-03

**Authors:** Chunxia Wen, Xinyue Ni, Mei Han, Yue Yu, Chuanqiang Liu, Yuan Zhang, Beining Zheng, Shouhua Feng

**Affiliations:** ^1^ State Key Laboratory of Inorganic Synthesis and Preparative Chemistry Jilin Provincial International Cooperation Key Laboratory of Advanced Inorganic Solid Functional Materials College of Chemistry Jilin University Changchun Jilin 130012 P. R. China; ^2^ College of Physics Jilin University Changchun 130012 P. R. China

**Keywords:** carrier separation efficiency, doping regulation, homogeneous photocatalysts, photocatalytic performance

## Abstract

Homogeneous matrix materials are considered to be a solid guarantee of site‐point charge transfer reactions because they are the main body of light absorption, photogenerated carrier separation, migration, and recombination processes. Elucidating the effect of carrier separation efficiency on catalytic performance is of great significance for overcoming the optimization obstacles of homogeneous matrix parts and providing new design strategies. In this study, TiO_2_ doped with Fe as the research object quantifies the carrier dynamics while trying to avoid large site and lattice changes. The direct correlation between carrier separation efficiency and photocatalytic performance with different doping content samples is clearly elucidated by carrier dynamic characterization results. Doping of 0.213 wt.% Fe exhibits the best catalytic performance, achieving CO yield of 35.12 µmol g^−1^ h^−1^. Especially, the femtosecond transient absorption spectroscopy demonstrates the defect level formed by doping Fe enhances the separation of photogenerated electrons. The clear relationship shown above, which is catalytic capacity mapping with carrier separation efficiency, rather than a linear dependence on sites and oxygen vacancies, fully demonstrates the great potential of simple doping strategies for homogeneous photocatalysts.

## Graphical abstract

A doping model of homogeneous photocatalysts of TiO_2_ is constructed to directly show the mapping relationship between carrier separation efficiency and photocatalytic performance. TRPL, KPFM, fs‐TAS measurements further quantify the carrier dynamics process. The photocatalytic performance of this series samples is dominated by the optimal conditions of carrier separation behaviour within a certain range, and does not form a linear dependence on the site, the oxygen vacancy.

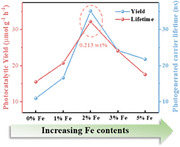

## 1 Introduction

For heterogeneous photocatalyst systems, the design of sites and their synergies with carriers, such as single atom dispersion,^[^ ^1^, ^2^, ^3^ ^]^ inducing surface isolator resonance^[^ ^4^, ^5^, ^6^ ^]^ and constructing Schottky barrier, has become the main research object.^[^ ^7^, ^8^, ^9^ ^]^ Nevertheless, the importance of the light‐matter interaction processes (such as photon absorption, photogenic carrier diffusion, and its lifetime extension) in the homogeneous host material also has been recognized in the exploration of improving performance. It has been reported that doping B in amorphous TiO_2_ to build defects effectively facilitated the use of light absorption by altering the energy band structure, thus favouring the photocatalytic process.^[^ ^10^ ^]^ A simple two‐atom site strategy has also been studied consisting of Cu‐N_4_ and C‐S‐C active portions modified on polymeric carbon nitride (Cu‐SAs/p‐CNS) to promote photogenerated carrier diffusion for improved photocatalytic performance.^[^ ^11^ ^]^ Researchers have reported the formation of homogeneous catalysts with high S vacancies by P gap‐filling doping CdS to modulate the energy band structure, thus extending its lifetime and effectively enhancing photocatalytic.^[^ ^12^ ^]^ Even back electron‐hole recombination has been studied in hematite photoanodes for water splitting.^[^ ^13^ ^]^ However, few reports can directly show the mapping relationship between carrier separation efficiency and photocatalytic performance by synthesizing various factors of the above light‐matter interaction process. Therefore, if the degree of carrier separation under specific regulatory strategies can be experimentally quantified, it will have important guiding significance to maximize its efficiency and thus induce high catalytic activity.

As the important role of enhanced electron‐hole separation on the increase of photocatalytic activity has been widely concerned, fine characterization methods that can further study the behavior of electron and hole separation have been increasingly applied to reveal the mechanism of photocatalytic reaction.^[^ ^14^, ^15^, ^16^, ^17^ ^]^ Among them, femtosecond transient absorption spectroscopy (fs‐TAS), is a powerful method to detect carrier dynamics, which makes it possible to study the rapid separation and transfer behavior of photogenerated carriers in materials due to its strong time resolution. For instance, Tse et al. investigated the photogenerated electron‐hole separation and carrier dynamics of the O_v_‐WO_3_ series of materials by TAS spectroscopy, and confirmed that O_v_ could produce electron‐trapping states and inhibit the direct complexation of photogenerated carriers.^[^ ^18^ ^]^ Jiang et al. also found that Co‐doped BNF led to effective phototropic interband transitions using TAS at 300 nm pump pulses. The study indicated that electron/hole recombination dynamics were greatly inhibited, and confirmed active role of Co atom in electron transfer.^[^ ^19^ ^]^ Additionally, Kelvin probe force microscopy (KPFM) can deepen the understanding of the electron‐hole spatial separation results by directly observing the surface sites of the material in the presence or absence of light. For example, Bi et al. detected the charge transfer process in Bi‐Bi_2_Sn_2_O_7_ catalyst by KPFM. The significant decrease in the surface potential under photoexcitation confirmed the electron enrichment on the surface of Bi‐Bi_2_Sn_2_O_7_.^[^ ^20^ ^]^ Furthermore, Ding et al. also used KPFM to observe the photogenerated charge migration path and surface potential changes of the prepared TiO_2_/FePS_3_ samples, revealing the photocatalytic mechanism of TiO_2_/FePS_3_ structure.^[^ ^21^ ^]^

Doping is a common strategy to adjust the bandgap, change the energy level structure, and control the aggregation state of oxygen defect.^[^ ^22^, ^23^, ^24^, ^25^ ^]^ Therefore, we tried to regulate the doping amount precisely in the main material to design in this paper. The relationship between the carrier separation efficiency and the photocatalytic performance is described based on a plot correlating carrier separation with doping variation. TiO_2_ was chosen as the host material due to strong catalytic ability and favorable electronic properties: 1) TiO_2_ obtained a wide bandgap (3.2 eV, anatase phase) and its optical absorption is very low in the visible range; 2) TiO_2_ could generate defect energy levels, which makes it have a strong potential for regulation carrier separation ability.^[^ ^26^, ^27^, ^28^, ^29^ ^]^ Fe was selected for doping based on structural properties of TiO_2_. The reasons for this choice include: 1) the ionic radius of Fe^3+^ is similar to that of Ti^4+^, preventing significant structural distortion; 2) Fe‐doped TiO_2_ can introduce Fe^3+^ shallow trap states. Despite existing studies on Fe‐doped TiO_2_,^[^ ^30^, ^31^, ^32^, ^33^ ^]^ few researches have optimized the doping concentration or quantified the carrier dynamics process in detail. The experimental procedure involved synthesizing a series of ideal occupying forms with varying Fe doping content to study the effect on the photocatalytic performance. The optimal catalyst with doping of 0.213 wt.% Fe showed excellent CO yield of 35.12 µmol·g^−1^·h^−1^ in CO_2_ photoreduction, which was 3.2 times higher than that of pristine TiO_2_. The dynamics were accurately characterized by TRPL, KPFM and TAS measurements, indicating that the defect level caused by the doping of Fe atoms leads to the longest electron capture lifetime, which promotes carrier separation. Through this doping model, it is fully proved that the great potential of carrier separation efficiency regulation on the performance of homogeneous catalysts.

## 2 Results and Discussion

### 2.1 Structure and Morphology

**Figure 1:**
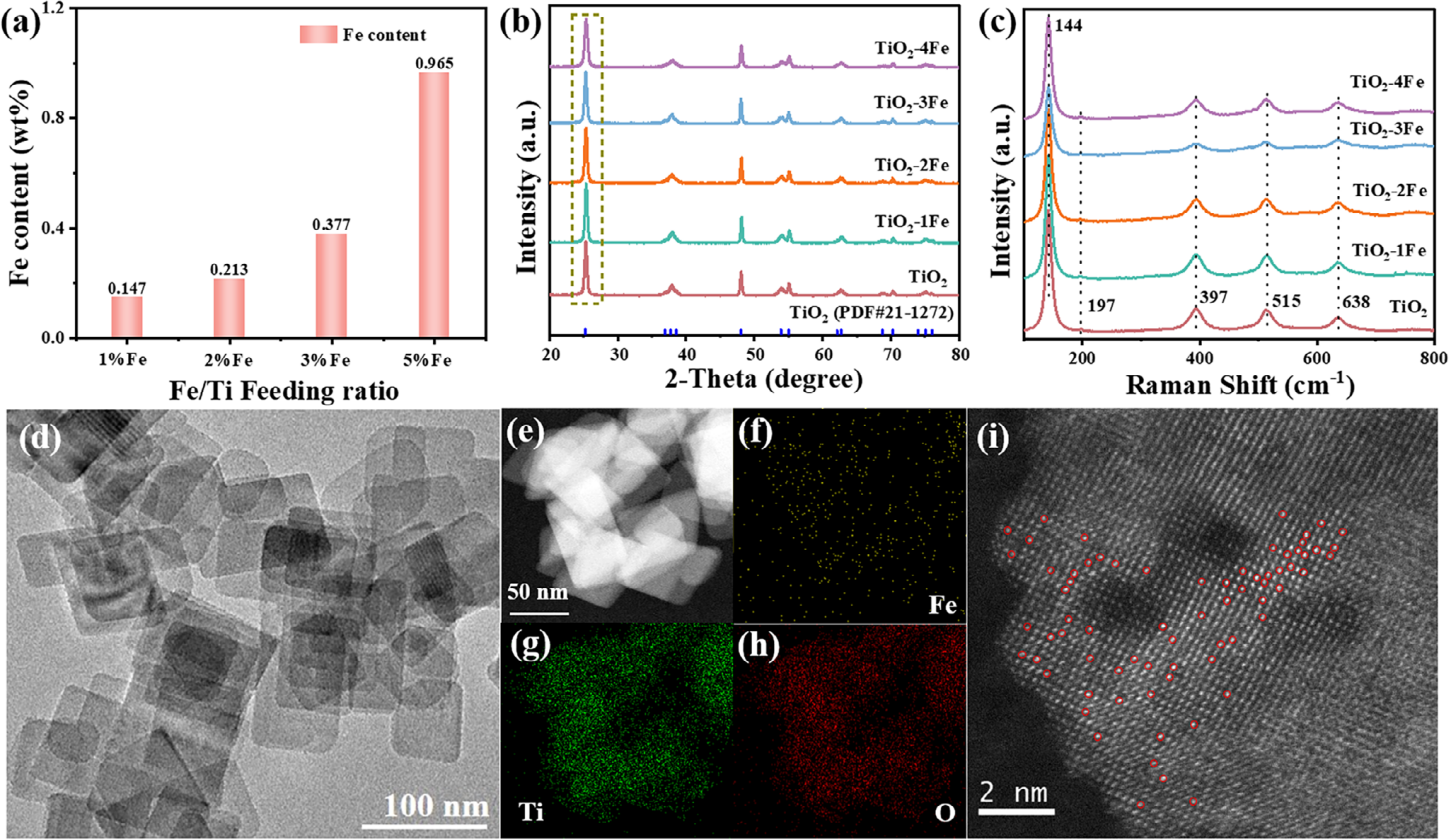
a) Fe content of TiO_2_‐Fe samples. b) XRD patterns of TiO_2_ and TiO_2_‐Fe samples. c) Raman spectra of TiO_2_ and TiO_2_‐Fe samples. d) TEM of TiO_2_‐2Fe. e–h) HAADF‐STEM and corresponding elemental mapping images of TiO_2_‐2Fe, and i) aberration‐corrected HAADF‐STEM image, where the spotlights are marked with red circles.

A series of Fe‐doped TiO_2_ nanosheets were synthesized via one‐step hydrothermal method. The iron content in the samples was measured using electron probe X‐ray micro‐analyzer (EPMA), which provided high quantitative analysis accuracy in **Figure** **1**a. The prepared samples are denoted as TiO_2_‐1Fe, TiO_2_‐2Fe, TiO_2_‐3Fe, and TiO_2_‐4Fe, with the mass fractions of Fe corresponding to 0.147, 0.193, 0.377, and 0.965 wt.%, respectively (Table S1, Supporting Information). It confirms that the Fe doping content increases gradually with the Fe/Ti the feed ratio. Figure 1b presents the X‐ray diffraction (XRD) patterns of pristine TiO_2_ and TiO_2_‐Fe samples. Clearly, all diffraction patterns were well‐indexed to anatase phase (PDF# 21–1272). A slight shift of the peak at 25.3° to a higher diffraction angle is observed, which could be attributed to the similarity in the ionic radius of Fe^3+^ (0.064 nm) and Ti^4+^ (0.068 nm). The substitution of Ti by Fe leads to a contraction of the TiO_2_ lattice and a slight decrease in diffraction peak intensity (Figure S1, Supporting Information). The surface chemical structure of TiO_2_ and TiO_2_‐Fe samples were further conducted using Raman spectroscopy in Figure 1c. All samples show similar peak patterns. Raman spectra correspond to characteristic peaks for the TiO_2_: three E_g_ models (144, 197, 638 cm^−1^), one B_1g_ model (397 cm^−1^), and (A_1g_+B_1g_) model ≈(515 cm^−1^), further confirming that the introduction of Fe does not destroy the initial TiO_2_ phase.^[^ ^30^, ^31^ ^]^ Compared to pristine TiO_2_, a decrease in peak intensity and gradual broadening of the peaks are observed in the Fe‐doped samples, suggesting that iron doping may activate the surrounding oxygen atoms, which leads to minor lattice distortion and the formation of lattice defects.^[^ ^34^ ^]^ Scanning electron microscopy (SEM) images of the TiO_2_‐Fe samples show similar morphology without significant differences (Figure S2, Supporting Information). The TiO_2_‐2Fe sample with the best photocatalytic activity was characterized by transmission electron microscopy (TEM) and high‐resolution transmission electron microscopy (HRTEM) (Figure 1d; Figure S3, Supporting Information). TiO_2_‐2Fe maintains similar nanosheet structures, compared with pristine TiO_2_ morphology (Figure S4, Supporting Information). No Fe nanoparticles are observed on the surface of TiO_2_‐2Fe nanosheets. To further confirm the introduction and distribution of Fe atoms, TiO_2_‐2Fe sample is analyzed using high‐angle annular dark‐field scanning transmission electron microscopy (HAADF‐STEM) as shown in Figure 1e–h. The elemental mapping spectra reveals that Fe, Ti, and O elements are evenly distributed throughout the TiO_2_‐2Fe framework. A high density of bright spots (highlighted in red circles) is observed in the aberration‐corrected HAADF‐STEM image as shown in Figure 1i. These bright spots are largely isolated and do not aggregate, confirming the introduction of Fe atoms.

**Figure 2:**
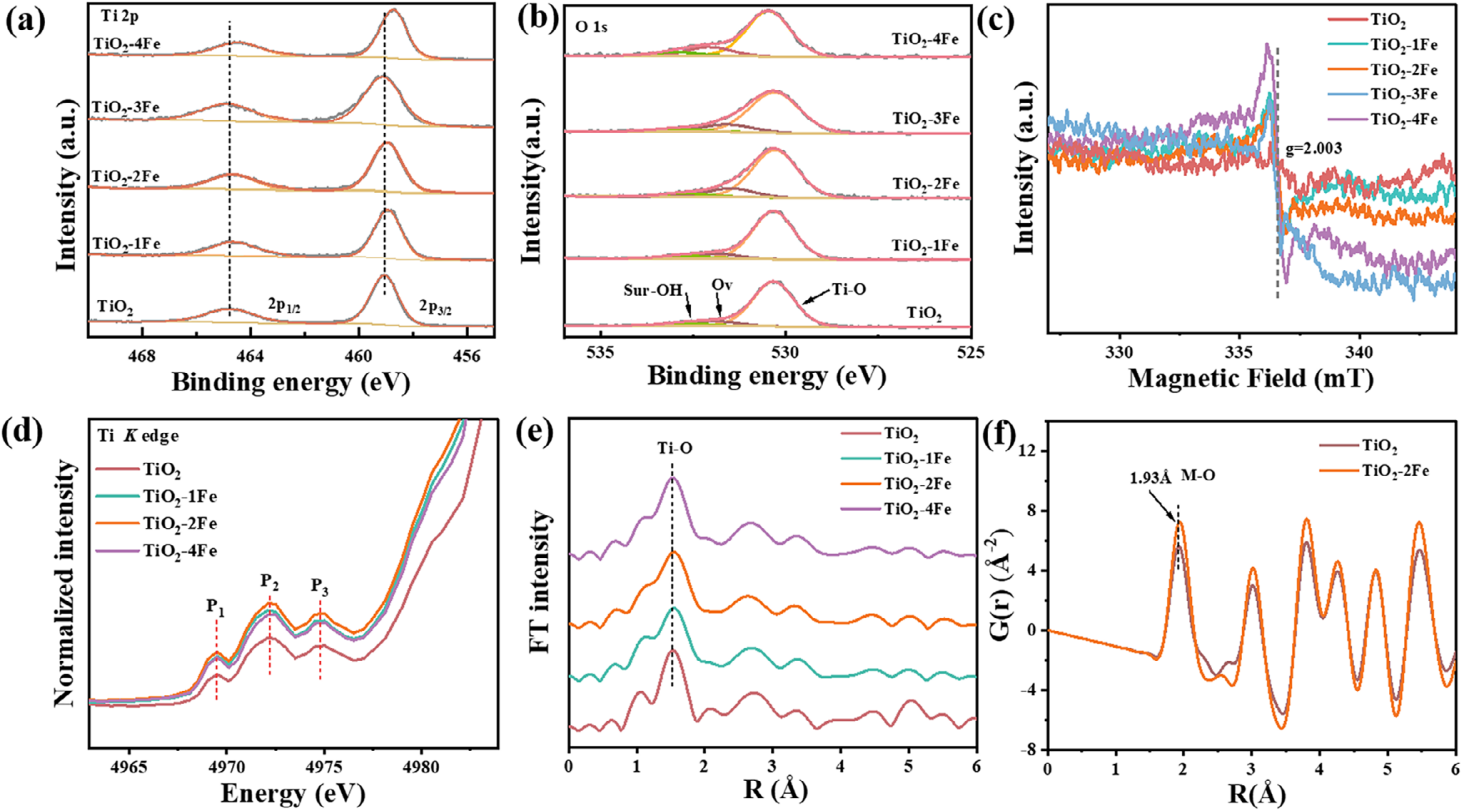
XPS spectra of TiO_2_ and TiO_2_‐Fe: a) Ti 2p, b) O 1s. c) EPR spectra. d) Ti *K*‐edge XANES spectra. e) Ti *K*‐edge FT EXAFS spectra of TiO_2_ and TiO_2_‐Fe samples in R space. f) Atomic distribution function (PDF) of TiO_2_ and TiO_2_‐2Fe samples.

X‐ray photoelectron spectroscopy (XPS) measurements were conducted to further characterize the chemical components and the electronic structure of the TiO_2_ and TiO_2_‐Fe samples. **Figure** **2**a shows two characteristic peaks with binding energy at 459.07 and 464.74 eV, which can be attributed to Ti 2p_3/2_ and Ti 2p_1/2_ in the Ti 2p spectra.^[^ ^35^, ^36^ ^]^ Notably, after Fe is introduced into TiO_2_, the Ti 2p_3/2_ and Ti 2p_1/2_ peaks shift toward lower binding energies, which confirms the reduction of the valence state of Ti and the formation of oxygen vacancies. Moreover, the XPS spectrum of O 1s component is resolved into three characteristic peaks at 530.33, 531.87, and 532.09 eV, which corresponds to lattice oxygen O_I_, oxygen vacancy O_II_, and surface chemisorbed oxygen O_III_, respectively in Figure 2b.^[^ ^37^, ^38^, ^39^ ^]^ The content of oxygen vacancy increases from 8.4% in pristine TiO_2_ to 15.35% in TiO_2_‐4Fe, indicating that Fe doping enhances the oxygen vacancy concentration (Figure S5 and Table S2, Supporting Information). Moreover, the electron paramagnetic resonance (EPR) tests reveal that there is no obvious signal for oxygen vacancy in pristine TiO_2_, while the TiO_2_‐Fe samples show a clear signal at *g* = 2.003, confirming that Fe doping could lead to the formation of oxygen vacancies in TiO_2_‐Fe samples (Figure 2c).^[^ ^40^, ^41^ ^]^ Additionally, the paramagnetic signal increases with the doped Fe content, indicating that higher doping leads to the formation of more oxygen vacancies, which is consistent with the O 1s XPS results. In the Fe 2p XPS spectrum of Fe‐doped series samples (Figure S6, Supporting Information), the binding energy of Fe 2p_3/2_ peak is detected ≈710.09 eV, confirming the introduction of Fe atom.^[^ ^39^, ^42^ ^]^

To probe the local and electronic structure of pristine TiO_2_ and TiO_2_‐Fe samples, Ti *K*‐edge X‐ray absorption near‐edge structure (XANES) spectra were recorded. The normalization was performed in the range of 4900–5300 eV for the Ti *K*‐edge. Three low‐intensity pre‐edge peaks are observed, corresponding to the transition of Ti from 1s to mixed 3d‐4p orbitals, as shown in Figure 2d.^[^ ^43^, ^44^, ^45^ ^]^ These features are closely related to the degree of mixing of the d‐p orbitals as well as to the distortion of the Ti centrosymmetric environment. Compared to pristine TiO_2_, the Fe‐doped samples exhibit enhanced P_2_ peaks, indicating that the introduction of Fe causes structural distortion at the TiO_6_.^[^ ^46^ ^]^ Interestingly, the Ti absorption edge of TiO_2_‐Fe shifts toward lower energies compared to TiO_2_, confirming the reduction of Ti cation valence states, which is consistent with Ti 2p XPS results (Figure S7, Supporting Information). The doping Fe effectively modulates the electronic occupation of the Ti orbitals. The Ti *K*‐edge Fourier transform (FT)‐EXAFS spectra were performed to further confirm the atomic coordination environment of doped samples. Notably, the peak at ≈1.5 Å in the first shell corresponds to the Ti─O bond in TiO_2_ (Figure 2e). The Ti─O bond distance in the first shell of the Fe‐doped samples was slightly larger than that in the TiO_2_, indicating that the introduction of Fe causes structural distortion slightly. Synchrotron atom pair distribution function G(r) analysis was performed for TiO_2_ and TiO_2_‐2Fe (Figure 2f). The main peak at about 1.93 Å corresponds to the metal‐oxygen (M─O) bond. The absorption signal of the M─O bond in the TiO_2_‐2Fe sample is stronger than that in the pristine TiO_2_, and the M─O bond distance is slightly larger, which is consistent with the results of EXAFS analysis.^[^ ^47^ ^]^

### 2.2 Photocatalytic Performance

**Figure 3:**
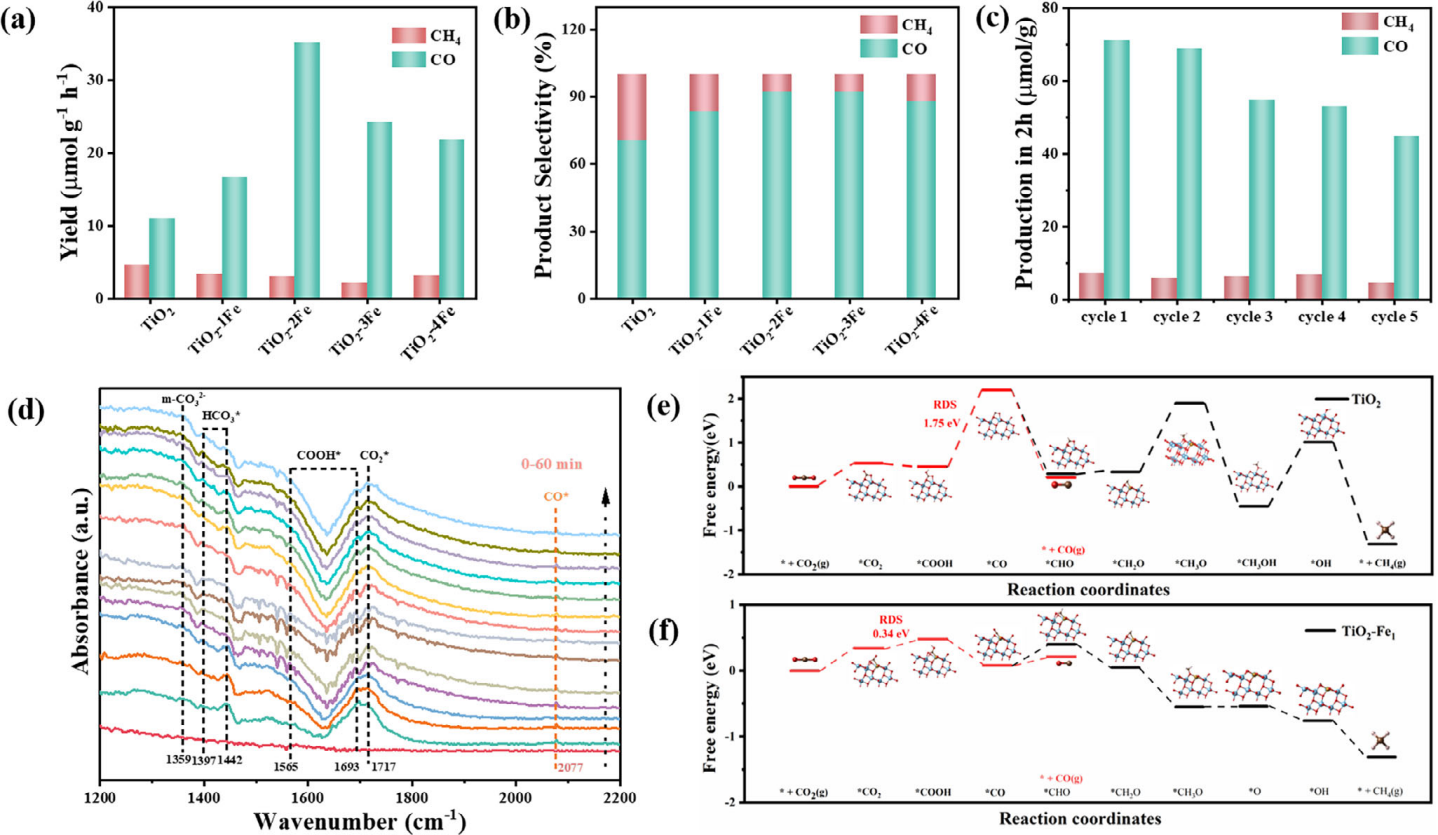
a) Photocatalytic CO_2_ reduction yield for TiO_2_ and TiO_2_‐Fe samples. b) Photocatalytic CO_2_ product selectivity. c) Cycling stability of TiO_2_‐2Fe. d) In situ FTIR spectra of TiO_2_‐2Fe. e,f) Calculated Gibbs free energy diagram for CO_2_ reduction for TiO_2_ and TiO_2_‐Fe_1_.

The photocatalytic CO_2_ reduction was carried out on a gas‐solid reaction apparatus under UV–visible irradiation. The conversion products of CO_2_ photocatalytic reduction for the synthesized samples are presented with CO and CH_4_ products in **Figure** **3**a. Among the samples, TiO_2_‐2Fe exhibits the best photocatalytic performance, with the CO conversion yield for TiO_2_‐2Fe > TiO_2_‐3Fe > TiO_2_‐4Fe > TiO_2_‐1Fe > TiO_2_. The catalytic performance of the samples does not increase linearly with Fe content. The yield of CO conversion for TiO_2_‐2Fe is 35.12 µmol·g^−1^·h^−1^, which is 3.2 times higher than that of pristine TiO_2_. A series of Fe‐doped samples exhibit enhanced catalytic performance compared to pristine TiO_2_, indicating that the introduction of Fe atoms improved the surface catalytic activity of TiO_2_ and facilitated the CO_2_ photocatalytic reduction. The photoreduction rate of CO_2_ to CH_4_ was relatively low with poor selectivity. The selectivity of the TiO_2_‐2Fe sample for CO_2_ reduction to CO is as high as 92%, compared to the 8% selectivity for CH_4_ in Figure 3b. Furthermore, TiO_2_‐2Fe, as the best performing sample, was tested for photocatalytic stability as shown in Figure 3c. Photocatalytic stability measurements were performed by extracting the reaction products for 2 h, followed by refilling the reactor with CO₂ for five cycles. The test datas are shown in Table S3 (Supporting Information). After five cycles of photoreaction, the CO yield remained at 63%, demonstrating good photocatalytic stability.

The TiO_2_‐2Fe sample was subjected to a long photocatalytic test for 12 h. After the reaction, the sample was collected and characterized by XRD and TEM in (Figures S8 and S9, Supporting Information). Compared to the initial TiO_2_‐2Fe sample, the reacted sample maintained high crystallinity and the pure anatase phase without impurities formed. Subsequently, controlled experiments were performed to verify the necessary of the product CO (Figure S10, Supporting Information). In the absence of light, there was no product, only trace amounts of CO was detected under conditions without water vapor, catalyst, and in an argon atmosphere. In order to precisely demonstrate the source of the CO product, ^13^CO_2_‐labeled isotope experiments were performed. The result of the mass spectrometry is shown in (Figure S11, Supporting Information). The dominant peak of ^13^CO (m/z = 29) was observed in the CC‐MS for TiO_2_‐2Fe sample.^[^ ^48^, ^49^ ^]^ This result confirms that the source of CO production originates from the photoreduction of CO_2_. Moreover, TiO_2_‐2Fe demonstrates excellent performance and stability, superior to previously reported TiO_2_‐based photocatalysts (Table S4, Supporting Information). Based on the stability test of TiO_2_‐2Fe and the comparison of samples before and after the reaction, the photocatalysis also shows good cycle.

### 2.3 Reaction Mechanism of Photocatalytic CO_2_ Reduction

The CO_2_ temperature‐programmed desorption (TPD) analysis was conducted to characterize the chemical adsorption of CO_2_ on TiO_2_ and TiO_2_‐2Fe catalysts (Figure S12, Supporting Information). Notably, TiO_2_‐2Fe exhibits two distinct peaks corresponding to weakly chemisorbed CO_2_ at 278 °C and strongly chemisorbed CO_2_ at 380 °C. The absorption intensities of these peaks are substantially higher than those observed for pristine TiO_2_. Additionally, the peak associated with strong chemisorption shifts to higher temperatures, indicating the formation of robust bonds between oxygen vacancies and the O atoms of CO_2_, which is in agreement with the XPS results.^[^ ^50^ ^]^ This evidence confirms that Fe incorporation enhances CO_2_ chemisorption on the catalyst, thereby promoting the activation of the reaction. To gain a deeper understanding of the CO_2_ photoreduction process, TiO_2_ and TiO_2_‐2Fe samples were characterized using in situ Fourier transform infrared spectroscopy (in situ FTIR) measurements to identify the reaction intermediates involved in the photocatalytic process (Figure 3d; Figure S13, Supporting Information). Figure 3d shows the in situ FTIR spectra recorded at 2 min intervals after introducing CO_2_ and water vapor for 30 min in the presence of UV–visible light. The in situ FTIR spectra of the TiO_2_‐2Fe sample differ significantly from those of pristine TiO_2_ sample. The signal at 1359 cm^−1^, corresponding to m‐CO_3_^2−^, is assigned to monodentate bicarbonate.^[^ ^51^, ^52^ ^]^ Two peaks at 1397 and 1442 cm^−1^ belong to the surface‐bound monodentate carbonate HCO_3_^−^.^[^ ^53^ ^]^ The presence of m‐CO_3_^2−^ and HCO_3_^−^ intermediates proved the adsorption of CO_2_ on the TiO_2_‐2Fe surface. The peak at 1717 cm^−1^ is attributed to the ^*^CO_2_ intermediate,^[^ ^54^ ^]^ indicating that CO_2_ adsorbed on the TiO_2_‐2Fe sample was more effectively activated. Additionally, ^*^COOH is detected on the peaks ≈1565 and 1693 cm^−1^, which is a key intermediate in the process of CO generation. The TiO_2_‐2Fe samples exhibited stronger absorption signals.^[^ ^55^ ^]^ Furthermore, the peak is detected at 2077 cm^−1^, corresponding to the ^*^CO intermediate. The ^*^CO intermediate signal is also stronger in the TiO_2_‐2Fe sample compared to the original TiO_2_.^[^ ^56^ ^]^ Notably, the intensity of the ^*^CO intermediate peak increased with irradiation time, suggesting that the photocatalytic reaction was continuous and that ^*^CO intermediates played a critical role in generating CO products.^[^ ^57^ ^]^

To further explore the charge transfer pathways and catalytic sites of TiO_2_‐Fe in the photocatalytic process, the in situ XPS was tested under visible light. The binding energies of Ti 2p_1/2_ shows positive shift under illumination (Figure S14, Supporting Information), indicating the loss of e^−^ and the decrease of the electron density around Ti 2p. Similarly, the BE of O 1s XPS peaks increases under light, and the electron density is reduced around O 1s. Conversely, for XPS of Fe 2p, the peak of Fe 2p shifts toward lower binding energy under illumination, indicating that the electron density around Fe 2p is elevated. Obviously, the above results show that the electron of TiO_2_ surface leads to electron loss under light and charge density aggregation at the Fe site, it can be seen that electron flow is transferred from TiO_2_ to Fe site. This suggests that the introduction of Fe sites facilitates the aggregation of photogenerated electrons, thereby enhancing the rate of photocatalytic product formation.

To further investigate the mechanism of photocatalytic reduction of CO_2_, we performed density‐functional theory (DFT) calculations. The crystal structures of pristine anatase titanium dioxide (101) facets and iron‐doped model (TiO_2_‐Fe_1_) were modeled, respectively (Figure S15, Supporting Information). Replacement of Ti^4+^ with Fe^3+^ may produce oxygen vacancies due to charge balance. The CO_2_ adsorption configuration of TiO_2_‐Fe_1_ was optimized by the coordination of Fe atoms and nearby O atoms with CO_2_ (Figure S16, Supporting Information). The CO_2_ adsorption energy of TiO_2_‐Fe_1_ increases 0.19 eV, compared to that of the pristine TiO_2_, which proves that doping Fe can promote the adsorption of CO_2_ (Figure S17, Supporting Information).^[^ ^32^ ^]^ Furthermore, the charge density difference of CO_2_ adsorption was performed on the model. The charge density between Fe site and C atom increases in the TiO_2_‐Fe_1_ model. In the original TiO_2_, atom C is connected to bridge O of TiO_2_, and the charge density decreases between the two atoms. Therefore, TiO_2_‐Fe_1_ is more conducive to the activation of CO_2_ molecules. Further calculation of the Bader charge transfer value at the catalytic site shows that the charge transfer of TiO_2_‐Fe_1_ is stronger than that pristine TiO_2_. The above calculation results show that Fe site also acts as an active site after the introduction of Fe atoms, promoting the rapid transfer of photoexcited electrons to the adsorbed CO_2_, and benefitting the formation of CO product. Combining with the in situ FTIR measurements and CO_2_‐TPD experiments, the Gibbs free energy diagram along with the corresponding optimized intermediate states for the reduction of CO_2_ to CO are shown in Figure 3e,f. For TiO_2_‐Fe_1_, CO_2_ is adsorbed on the surface sites of TiO_2_‐Fe_1_ via double coordination, with Fe atoms and oxygen vacancies serving as the active sites for adsorption, Specifically, the C atom binds to the Fe site, while the O atom attaches to the oxygen vacancy coordinated with the neighboring Ti atom. Following structural optimization, the dissociated proton from H_2_O binds to the O of CO_2_, forming the ^*^COOH intermediate.^[^ ^58^ ^]^ Subsequently, the ^*^COOH intermediate accepts a proton, leading to the formation of ^*^CO through the desorption of H_2_O, which occurs in a linear configuration. Finally, the desorption of the ^*^CO intermediate yields the CO product. Notably, the generation of ^*^COOH is the rate‐determining step of the process with energy barrier of 0.34 eV. In contrast, for pristine TiO_2_, the bis‐coordinated adsorption configuration of CO_2_ involves the C and O atoms binding to the O and Ti sites on the TiO_2_ surface, respectively. The rate‐determining step is the formation of ^*^CO intermediates with a ΔG value of 1.75 eV. Significantly, for TiO_2_‐Fe_1_, the ΔG value of the formation of ^*^CO intermediates is much lower than that of pristine TiO_2_, leading to an accelerated reaction rate of CO generation. In order to explain the difference of the CO adsorption energy barrier, we further calculated the CO adsorbed the molecular charge densities for the valence band maximum (VBM) of the original TiO_2_ and TiO_2_‐Fe_1_ models (Figure S18, Supporting Information). The Fe 3d orbitals contribute to the composition of the top orbital of the VBM with the introduction of Fe atom. The formation of oxygen vacancies also promotes the adsorption of the ^*^CO intermediate, with the adsorption energy of ^*^CO in TiO_2_‐Fe_1_ (−0.13 eV) being significantly lower than in pristine TiO_2_ (1.99 eV). This finding is consistent with the changes in ^*^CO intermediates detected by in situ FTIR. Regarding the intermediates for CH_4_ formation, thermodynamic calculations indicate that the Δ*G* value of ^*^CHO intermediate is higher than that of the ^*^CO intermediate in the CO_2_ reduction pathway of TiO_2_‐Fe_1_. This higher energy for ^*^CHO formation hinders the production of CH_4_. Therefore, TiO_2_‐Fe_1_ exhibits better product selectivity for CO compared to pristine TiO_2_. For the intermediates formation of CH_4_ product, the thermodynamic perspective indicated that Δ*G* of the intermediate ^*^CHO was higher than ^*^CO intermediate in the CO_2_ reduction path of TiO_2_‐Fe_1_, which hindered the formation of CH_4_ products. Therefore, TiO_2_‐Fe_1_ obtained better product selectivity for CO product, compared to the original TiO_2_. The above results show that the introduction of Fe atom markedly decreases the energy barrier of ^*^CO intermediates, thereby enhancing the catalyst's efficiency in improving the activity and selectivity of CO_2_ reduction.

### 2.4 Optical Absorption Properties and Photogenerative Carrier Dynamics

**Figure 4:**
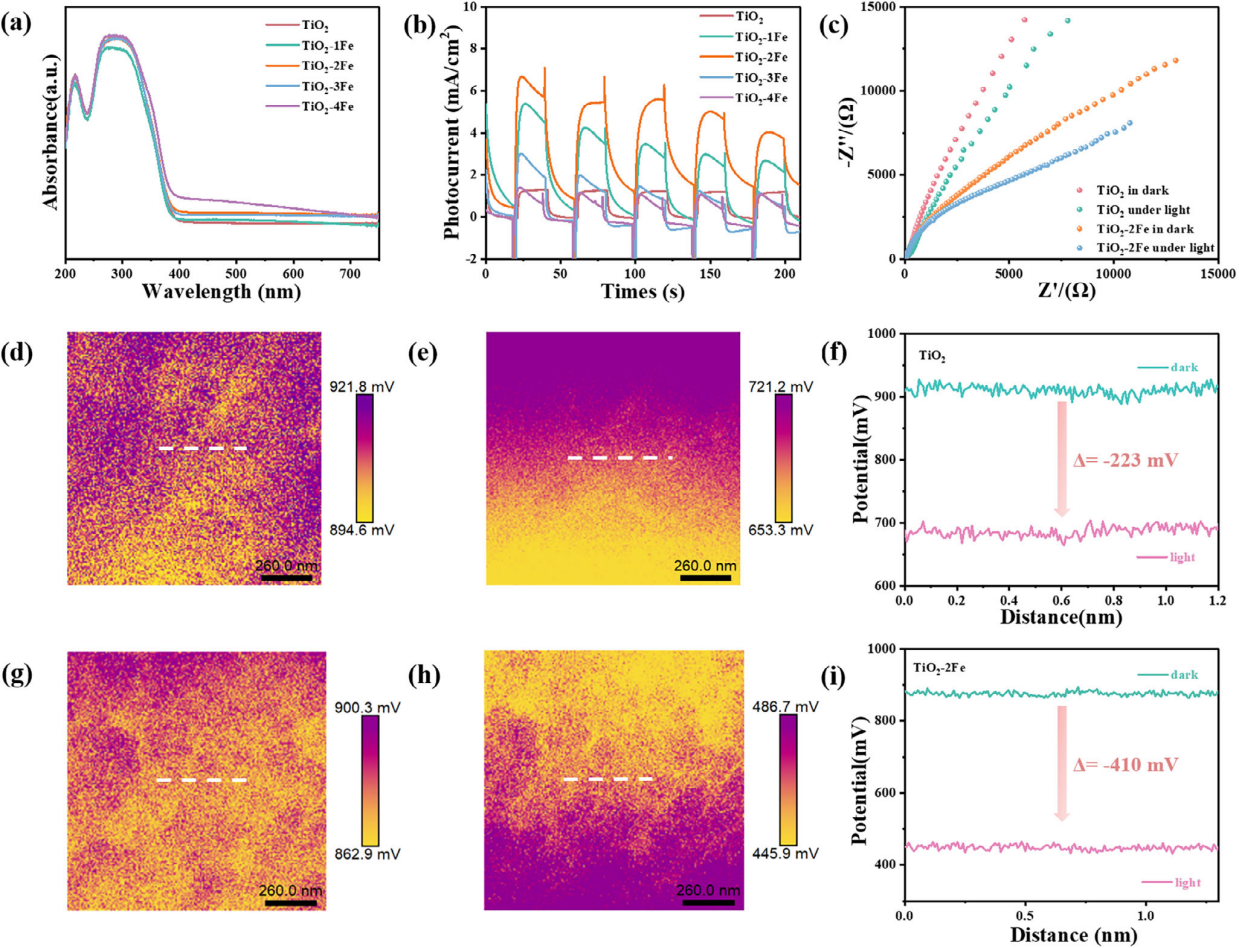
a) UV–vis spectra. b) Transient photocurrent responses. c) EIS spectra for TiO_2_ and TiO_2_‐2 Fe samples under dark and light. d–i) KPFM pictures of TiO_2_ and TiO_2_‐2Fe: d,g) Corresponding the surface potential distribution under darkness and e,h) under light. f,i) The line‐scanning surface potential of TiO_2_ and TiO_2_‐2 Fe, respectively.

To investigate the light absorption properties on the photocatalytic performance of Fe‐doped TiO_2_ samples, the band structure of the samples was characterized. **Figure** **4**a records the UV–vis spectra of the prepared samples. The original TiO_2_ shows an ultraviolet absorption edge of ≈380 nm.^[^ ^30^, ^59^ ^]^ The absorption intensity of Fe‐doped series samples increases in the visible range of 400–800 nm, which is due to the narrowing of the bandgap caused by Fe‐doped series samples. The reason for the narrowing of the bandgap can be attributed to the d‐d conversion of electrons between Fe^3+^ and Ti 3d orbitals after the introduction of Fe atoms, which can capture photogenerated electrons.^[^ ^60^ ^]^ The defect level caused by oxygen vacancies can be used as an electron trap, which promotes the narrowing of the bandgap and enhances light absorption. Furthermore, the bandgap *E_g_* for TiO_2_ and TiO_2_‐2 Fe is 3.09 and 2.94 eV, respectively, calculated through the Tauc plots of (*αhv*)^1/2^ versus photo energy (*hv*) in Figure S19 (Supporting Information).^[^ ^59^ ^]^ According to XPS valence band spectra, the valence band edge positions of TiO_2_ and TiO_2_‐2Fe were examined. The specific spectra showed that the edge positions of TiO_2_ and TiO_2_‐2Fe samples were 2.94 and 2.89 eV, respectively (Figure S20, Supporting Information). The valence band potential showed a decreasing trend. According to the formula *E*_VB_ (vs NHE) =* φ* + *E*_VB, XPS_ – 4.44 eV, the VB edge potential is converted to the potential relative to the normalized hydrogen electrode (NHE), *φ* is the work function of the instrument, and the value is 4.35 eV.^[^ ^61^ ^]^ Therefore, the VB edge potentials of TiO_2_ and TiO_2_‐2Fe relative to NHE are 2.85 and 2.77 eV, respectively. Combined with the bandgap *E*_g_, the conduction potential is calculated by the *E*_CB_ = *E*_VB_‐*E*_g_ formula, which is −0.24 and −0.17 eV, respectively. The calculated band structure is shown in Figure S21 (Supporting Information). The band structure of the original TiO_2_ and the Fe doped TiO_2_‐2Fe sample more clearly indicates that the bandgap of the doped TiO_2_‐2Fe sample was narrowed, which further explains the reason for the improved spectral utilization. It can be seen that both CB and VB potentials can realize the redox process, which is thermodynamically feasible, according to the energy band diagram of the catalyst. In conclusion, the introduction of Fe atoms in TiO_2_ provides a favorable prerequisite for photocatalytic CO_2_ reduction.

Photo‐electrochemical characterization was carried out to further study the photoresponse ability of pristine TiO_2_ and TiO_2_‐Fe. The transient photocurrent response of the pristine TiO_2_ and TiO_2_‐Fe samples are listed after several on‐off cycles under xenon lamp irradiation in Figure 4b. Compared with the pristine TiO_2_, the samples with Fe doped TiO_2_ show a significant increase in photocurrent. TiO_2_‐2Fe shows the highest photocurrent density, indicating that the introduction of the moderate Fe amount in TiO_2_ can act as a charge medium and enhance the photoresponse ability.^[^ ^42^ ^]^

In addition to charge separation, charge transfer efficiency was also an important factor affecting the activity. The EIS results mainly reflected the charge‐carrier transfer kinetics. In general, the smaller the radius of the Nyquist curve, the faster the Faraday charge transfer at the interface. As shown in Figure 4c, the semicircle of TiO_2_‐2Fe is much smaller than original TiO_2_, showing that the electron transfer is effectively enhanced after Fe doping. In addition, the semicircle diameters of the sample are smaller in the light than those in the dark, indicating that the electron transfer was effectively improved under light conditions, and the photogenerated carriers’ migration speed is the highest. In order to further analyze the mechanism of photogenerated carrier transfer, the charge accumulation of Fe‐doped samples was measured by KPFM.^[^ ^62^, ^63^ ^]^ The surface potential diagram of the pristine TiO_2_ and TiO_2_‐2Fe samples under dark (Figure 4d,g) and light conditions (Figure 4e,h) were measured. The surface potential detected by the original TiO_2_ under photoexcitation conditions is reduced, and the surface potential difference is reduced by 223 mV compared to the surface potential under dark conditions in Figure 4f. The surface potential of TiO_2_‐2Fe sample decreases significantly under light excitation, and the surface potential difference is reduced by 410 mV in Figure 4i, significantly higher than that of the original TiO_2_. However, with the increase of Fe doping content, the surface potential difference of TiO_2_‐3Fe and TiO_2_‐4Fe is 231 and 102 mV under light irradiation and dark conditions, respectively in Figure S22 (Supporting Information). The increase of doping content will decrease the surface potential difference. The results are consistent with transient photocurrent, showing that TiO_2_‐2Fe sample obtains the best photogenerative carrier dynamics and photocatalytic performance. On the contrary, excessive doping not only diminishes light responsiveness but also adversely affects catalytic performance. The result clearly reflects that there is a tight mapping relationship between the photogenerated carrier dynamics and the photocatalytic performance.

**Figure 5:**
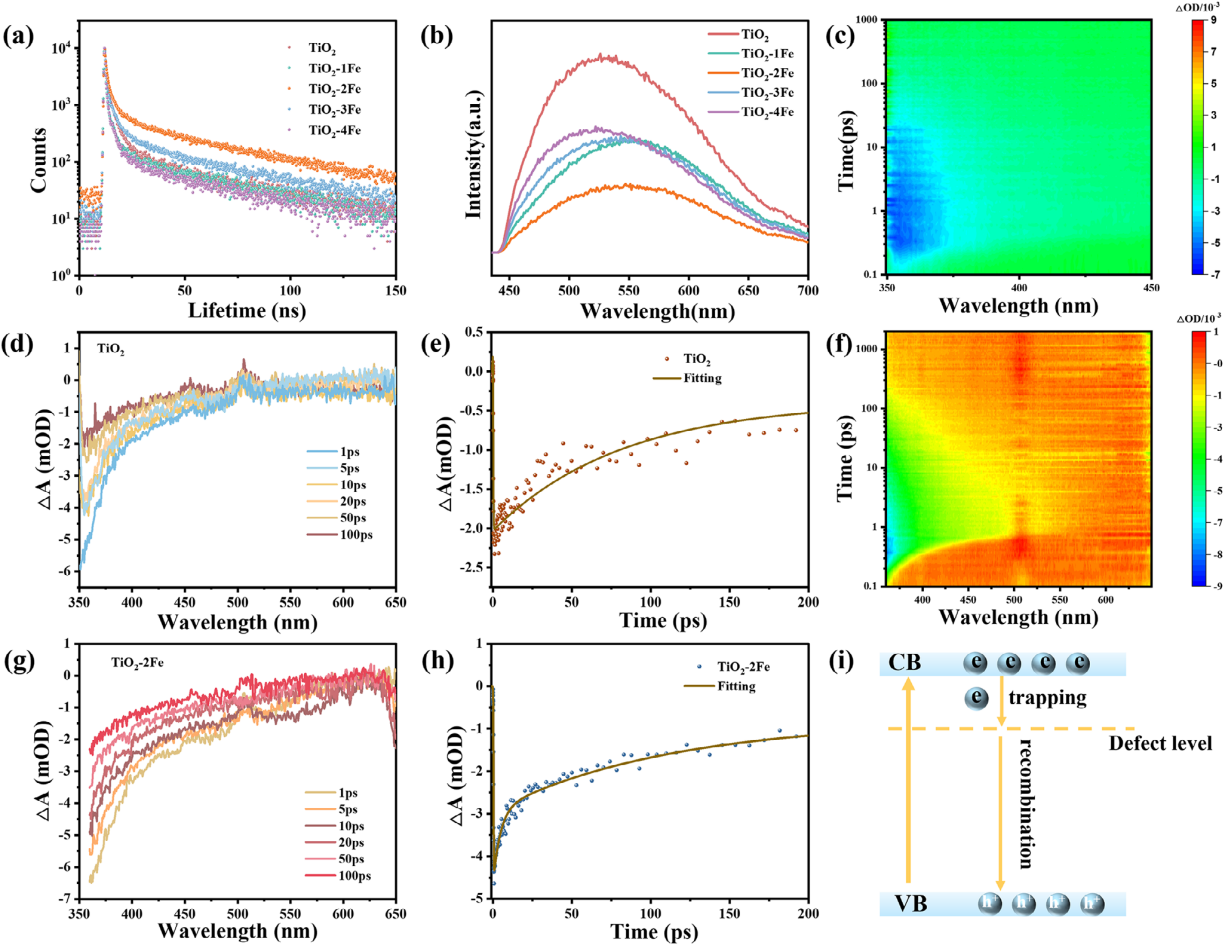
a) PL spectra. b) TRPL spectra. c–h) fs‐TAS monitors the photogenerated carrier dynamics for TiO_2_ and TiO_2_‐2Fe: c,f) 2D pseudo color pictures. d,g) Transient fs‐TA spectra under different probe delays. e,h) fs‐TAS kinetics decay profile probed at 380 nm. i) Schematic illustration for photoinduced dynamic.

To further clarify the nature of the effect of photogenerated carrier dynamics on the photocatalytic performance, we carried out characterization of transient behavior of photogenerated carriers.^[^ ^64^ ^]^ Photoluminescence (PL) (excitation wavelength λ = 365 nm) spectra show that the intensity of TiO_2_‐2Fe is the weakest, compared with other samples, indicating effective charge carrier separation (**Figure** **5**a). In addition, the lifetime of the photogenerated carrier was measured using time‐resolved spectroscopy based on the excitation absorption edge of the sample (excitation wavelength λ = 500 nm) in Figure 5b. The average carrier lifetime of the prepared samples obtained by fitting is shown in Table S5 (Supporting Information). The average carrier lifetime (*τ_ave_*) of all samples (TiO_2_‐2Fe > TiO_2_‐3Fe > TiO_2_‐1Fe> TiO_2_‐4Fe > TiO_2_) increases from 15.45 ns for TiO_2_ to 32.22 ns. This may be the optimal charge transport channel caused by the appropriate content of Fe doping, which promotes the photoinduced charge‐hole separation and inhibits its recombination. So the photogenerated carrier has the longest average lifetime for TiO_2_‐2Fe.

To further more accurately characterize the electron‐hole separation and carrier dynamics of TiO_2_ and TiO_2_‐Fe samples, the femtosecond transient absorption spectroscopy (fs‐TA) measurements were performed under 340 nm laser flash photolysis for TiO_2_ and TiO_2_‐Fe samples. The pseudo color TA plots of TiO_2_ and TiO_2_‐Fe samples are shown in (Figure 5c,f; Figure S20a,d, Supporting Information), respectively. Meanwhile, fs‐TA spectra were measured with probe delays varying from 1 to 100 ps (Figure 5d,g; Figure S20b,e, Supporting Information). The negative signal corresponds to the hole bleaching signal in the TiO_2_ valence band.^[^ ^65^, ^66^ ^]^ After the doping Fe atoms, the hole bleaching signal appears to increase than that of TiO_2_. This indicates that electrons are injected into Fe atom from TiO_2_, thus inhibiting the photogenerated carrier recombination. Notably, TiO_2_‐2Fe demonstrates the strongest absorption band. However, as excess iron content increases, there is a corresponding decrease in absorption signals. This observation indicates that TiO_2_‐2Fe sample facilitates rapid electron‐hole separation more effectively than others.

Furthermore, the decay curves of TiO_2_ and TiO_2_‐Fe were recorded and fitted by a two exponential function to investigate the kinetics of photogenerated electrons (Figure 5e,h; Figure S23c,f, Supporting Information). For TiO_2_ (*τ*_1_ = 1.95 ps, *τ*_2_ = 52.7 ps), the short lifetime *τ*_1_ is attributed to the free electron in the TiO_2_ conduction band, and the lifetime *τ*_2_ is attributed to the recombination time between the electron and the hole in the valence band (Table S6, Supporting Information).^[^ ^67^ ^]^ For TiO_2_‐Fe samples, *τ*_1_ is attributed to the extended recombination lifetime of the photoinduced electron‐hole pair, and the longer lifetime *τ*_2_ is attributed to the electron capture process caused by the defect state. Compared to other Fe doped samples, TiO_2_‐2Fe sample obtains the longest electron capture lifetime (*τ*_2_ = 139 ps), while *τ*_2_ decreases with the introduction of excess Fe. The reason for the decrease of the carrier separation efficiency may be the existence of deep trap state caused by excessive doping. The above time‐resolved transient results indicate that the enhancement of photogenerated electrons and hole separation induced by Fe atoms because the lattice site of Fe doping and the formed oxygen vacancy can both act as electron traps (photoexcited electrons transfer to Fe atoms, O_v_ forms a defect level, and can also capture photogenerated electrons), and the two synergistically enhance the separation of photogenerated electrons. At the same time, the separation efficiency controlled by doping is optimal and can be induced by proper doping. Based on kinetic analysis, doping‐induced photogenerated carrier separation mechanism with two relaxation processes is shown in Figure 5i. This fully shows that the charge separation efficiency in semiconductor materials is an important factor leading the catalytic performance. Additionally, the optimal separation efficiency requires a clear design strategy to guide the specific synthesis and regulation of homogeneous materials.

### 2.5 Band Structure Calculation

**Figure 6:**
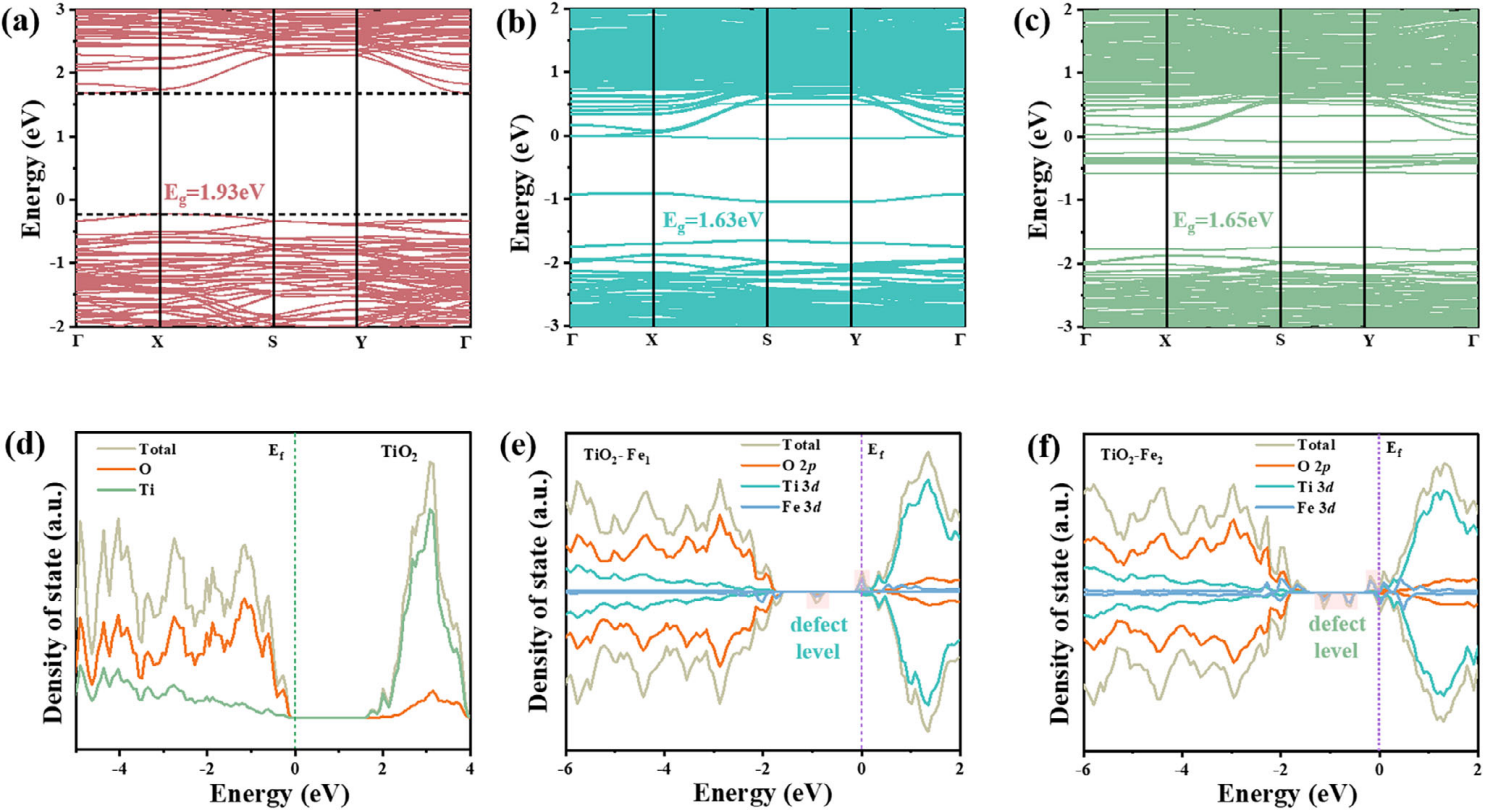
a–c) Band structure for TiO_2_, TiO_2_‐Fe_1,_ and TiO_2_‐Fe_2_, respectively. d–f) calculated density of states (DOS) for TiO_2_, TiO_2_‐Fe_1_ and TiO_2_‐Fe_2_, respectively.

In order to further explain the influence of the introduction of Fe atoms and the formation of oxygen vacancy on the carrier separation efficiency, the density of states (DOS) and band structure of Fe‐doped homogeneous materials were calculated in **Figure** **6**a–c (introducing one Fe atom and two Fe atom model in Figure S24, Supporting Information). the introduction of Fe atoms results in the appearance of defect energy levels in the energy level diagram (in the middle of Ti‐3*d* and O‐2*p* states) caused by Fe atoms and oxygen vacancies, in agreement with the results of experimental tests. The distribution of the number of defect energy levels increases with the increase of Fe content. Figure 6d–f calculates the density of states for TiO_2_, TiO_2_‐Fe_1_ and TiO_2_‐Fe_2_. The results also show this variation around the Fermi level, specifically for the Ti, O, and Fe hybridization appearing as the same attributed electronic states. The conduction band (CB) minima in both samples are primarily dominated by Ti 3*d* states, while the valence band (VB) maxima are mainly attributed to O 2*p* states (Figures S25–S27, Supporting Information). Fe doping leads to a decrease in the bandgap (TiO_2_ for 1.9 eV, TiO_2_‐Fe_1_ for 1.63 eV, and TiO_2_‐Fe_2_ for 1.65 eV), which is consistent with the UV–vis result. Further calculations of the fractional electronic density of states of TiO_2_‐Fe_1_ and TiO_2_‐Fe_2_ indicate that the 3d orbitals of Fe mainly contribute to the level between VB and CB (Figures S23–S27, Supporting Information). This suggests that photogenerated electrons are more likely to migrate toward the Fe site. The aforementioned results not only elucidate the reasons behind the variations in conductivity but also clarify the formation of intermediate energy levels during the migration of photo‐excited charge carriers. This will promote the photogenerated carrier separation. When a certain amount of iron atoms is doped, the density and distribution of defect energy levels enhance the recombination of photogenerated charge carriers. Consequently, this reduces the optimization effect, which is consistent with the previously discussed photogenerated carrier dynamics characterization results.

## 3 Conclusion

In conclusion, a homogeneous catalyst (Ti─O─Fe) was synthesized by selecting appropriate substrates and dopant atom, and minimizing the impact of lattice distortions, sites, and defects. Strong evidence obtained from TRPL, KPFM, TAS, and DFT calculations showed the correspondence between carrier dynamics and doping content. The study also elucidated the regulation of these dynamics in terms of energy level structure. The constructed doping model successfully quantified the correlation between carrier separation and photocatalytic performance. The photocatalytic activity was mainly determined by the optimal carrier separation conditions within a specific range and did not exhibit a linear dependence on the site concentration or oxygen vacancy levels (the optimal sample containing 0.213 wt.%Fe). This work highlights the critical role of carrier separation as a kinetic parameter, and enhances the understanding of energy level structure modulation in homogeneous catalysts. This modulation includes not only doping, but also the creation of defects, alteration of thickness, and post‐treatment strategies. Finally, the findings are expected to provide valuable insights into the design of complex non‐homogeneous catalysts.

## 4 Experimental Section

### Synthesis of TiO_2_ and TiO_2_‐Fe Samples

TiO_2_‐Fe samples were synthesized as follows. In a typical procedure, a specific amount of Fe(NO_3_)_3_·9H_2_O was added into Ti(OBu)_4_ (5 mL) and HF (0.6 mL, 40 wt.%). After stirring for 0.5 h, the solution was transferred into a Teflon‐lined autoclave (25 mL) and heated at 200 °C for 24 h. After cooling to room temperature, the precipitates were collected by centrifugation and washed with ethanol and ultrapure water for six times. Finally, the as‐prepared products were dried at 60 °C under a vacuum for 12 h. The synthesized TiO_2_‐Fe samples were labeled as TiO_2_‐1Fe, TiO_2_‐2Fe, TiO_2_‐3Fe and TiO_2_‐4Fe with actual iron content 0.147, 0.213, 0.377 and 0.965 wt.%, respectively. The TiO_2_ sample was synthesized using the same method without Fe(NO_3_)_3_·9H_2_O.

### Photocatalytic Tests for CO_2_ Reduction

Photocatalytic reduction CO_2_ was tested in a customised 180 mL Perfectlight PQ256 offline photocatalytic reactor. The reactor has two vents at the top for CO_2_ entry and exit. Two sampling ports were located below the reactor to collect liquid and gas phase products. The test was carried out under the condensate cycle. A Pls‐SXE300UV Xe lamp was used as the light source, and 2 mg sample was dispersed in 2 mL ultra‐pure water and sonicated for 20 min. The resulting dispersion was spread evenly across the bottom of the reactor. The sample was then dried in a vacuum oven at 60 °C. Once dry, the operation method was to control the flow rate of CO_2_ by means of a gas flowmeter. CO_2_ gas enters the quantitative 1mL pure water at the flow rate of 20 mL min^−1^, and was connected to the air inlet of the reactor, so that CO_2_ gas and water vapor are filled into the reactor with a 30 min to ensure a fixed ratio of water vapor and CO_2_. The reactor was placed under Xe lamp and condensate was turned on. The reaction proceeded for 2 h. At the end of the reaction, the gas from the reactor was collected in a 200 mL aluminium foil bag. Gas chromatography was performed using a Shimadzu GC‐2014C gas chromatograph. The gas chromatograph was equipped with a Thermal Conductivity Detector (TCD) and two Hydrogen Flame Ionization Detectors (FIDs) with built‐in methane converters. FID was equipped with Agilent HP‐Plot/Q and Agilent DB‐WAX columns. This setup enabled the simultaneous detection of CO, CH_4_, H_2_, and other olefin products.

### Photoelectrochemical Measurements

Transient photocurrent response spectra and electrochemical impedance spectra (EIS) were recorded by CHI‐760E electrochemical workstation. A three‐electrode system was employed, consisting of a working electrode (sample spin coated on FTO glass), a counter electrode (Pt sheet), and a reference electrode (saturated Ag/AgCl). The electrolyte was 0.5 m Na_2_SO_4_ solution. To prepare the working electrode, 5 mg of the sample was added to 1 mL ethanol and 10 µL naphthol, followed by ultrasonic dispersion for 30 min. The electrode was irradiated with a 300 W Xe lamp. EIS measurements were conducted at an AC voltage of 10 mV, with a frequency range from 0.1 to 1000 Hz.

## Conflict of Interest

The authors declare no conflict of interest.

## Author Contributions

C.W. wrote the manuscript. S.F. and B.Z. led the whole project. X.N. carried out theoretical calculations. Y.Y. measured the IMPE. Y.Z. and M.H. revised manuscript. C.L. measured the KPFM.

## Supporting information



Supporting Information

## Data Availability

The data that support the findings of this study are available from the corresponding author upon reasonable request.
