# Supplementary material for: The Function of Photocatalytic Performance and Carrier Separation Efficiency Tuned by Doping Content in Homogeneous Photocatalysts
Source: Adv Sci (Weinh). 2025 Apr 3;12(25):2501026. doi: 10.1002/advs.202501026 (PMC12225004; doi:10.1002/advs.202501026)
Supplement: Supplementary file 1 — Supporting Information [file ADVS-12-2501026-s001.docx]

**The Function of Photocatalytic Performance and Carrier Separation Efficiency Tuned by Doping Content in Homogeneous Photocatalysts**

Chunxia Wen ^a^, Xinyue Ni ^a^, Mei Han ^a^, Yue Yu ^b^, Chuanqiang Liu ^a^, Yuan Zhang ^a^, Beining Zheng ^b, *^ and Shouhua Feng ^a, *^

^a^ State Key Laboratory of Inorganic Synthesis and Preparative Chemistry, Jilin Provincial International Cooperation Key Laboratory of Advanced Inorganic Solid Functional Materials, College of Chemistry, Jilin University, Changchun, Jilin, 130012 P. R. China

^b^ College of Physics, Jilin University, Changchun 130012, P. R. China

E-mail: zhengbeining@jlu.edu.cn (B. Zheng), shfeng@jlu.edu.cn (S. Feng).

**Characterization**

Phase compositions of the samples were were performed using X-ray diffraction (XRD, BRUKER, D8 Advance) filtered Cu Kα radiation (λ=1.5418 Å). Scanning electron microscope (SEM, FEI, Helios NanoLab 600i Dual Beam System) and high-resolutiontransmission electron microscopy (HRTEM, FEI, Tecnai G2 S-Twin F20) were obtained to characterize the surface morphology and structure of the samples . X-ray photoelectron spectrum (XPS, Thermo Fisher Scientific, ESCALAB 250) was recorded using Al Kα line as the X-ray source. UV–vis absorption spectra were performed using a Shimadzu UV-4100 spectrophotometer. Raman spectroscopy was measured with INVIA of British Renishaw Company. In *situ* Fourier transform infrared spectra was recorded through Fourier transform infrared spectrometer (FT-IR, Bruker, VERTEX 80V). Photoluminescence spectroscopy was performed with fluorescence spectrophotometer (PL, Edinburgh Instruments, FLS920) and a CHI 760E workstation (CH Instruments) was used for the recordingof photocurrent. The near-edge structure spectra of Ti,O elements in the sample were characterized by BL12B-a station (China Synchrotron Radiation Laboratory, Hefei, China). Kelvin Probe (KPFM) images were recorded using bruker Dimension Icon. The fs-TA spectrum were measured in the combined utilization of the Femtosecond Laser System (Coherent) and the Helios Pump-Probe System (Ultrafast Systems LLC) with 340 nm laser pulses.

**Materials**

Tetra-n-butyl titanate (Ti(OBu)4), iron nitrate trihydrate (Fe(NO3)3·9H2O), hydrofluoric acid (HF, 40 wt.%), The Ti(OBu)4 and Fe(NO3)3·9H2O were of analytical grade. Ultra-pure water was used in all experiments.

**Transient Absorption Spectrum Measurements**

The femtosecond pulsed laser generated by the titanium-doped sapphire laser is divided into two beams after passing through a beam splitter: the first beam is the pump light, which first passes through the Faraday Isolator (FI) and is then chopped by the Acousto-Optical Modulator (AOM). The first beam is pumped, first passes through a Faraday isolator (FI) and then is chopped by an acousto-optic modulator (AOM). The beam is expanded by a beam expander (BE) and then passes through a half-wave wavelength and a polariser to adjust the light intensity and polarisation. The other beam as the detection light is expanded by the optical parametric oscillator (OPO) after tuning, and then the optical range difference between the pump light and the detection light is adjusted by the delayer. The pump light and the probe light are summed up in a dichroic mirror (DM). After passing through the 4f optical system, they are converged to the sample surface by an armed guard. Scanning imaging of the whole sample at different locations is achieved.

**In-*situ* FTIR** **Measurements**

In-*situ* Fourier transform infrared spectroscopy experiments were recorded using BRUKER-70 Fourier transform infrared spectrometer. Lay the TiO_2_-2Fe and TiO_2_ samples in a circular sample tray. After pretreatment in argon atmosphere for 0.5 hour to remove moisture and impurities, the back-bottom spectrum was obtained. After passing water vapor and CO_2_, adsorb for 0.5 hour. The xenon lamp source was then illuminated on the sample pool and the spectrum was measured every two minutes for one hour.

**Computational Tetails**

The density function theory (DFT) calculations were performed using Vienna ab initio simulation package (VASP) with the projector augmented-wave (PAW) method. ^[1-3]^ The generalized gradient approximation (GGA) functional parametrized by Perde Burke-Ernzerh of (PBE) was employed to describe the exchange correlation potential. ^[4]^ The DFT-D3 method was chosen for van der Waals correction.^[5]^ A plane-wave energy cutoff of 500 eV was used. The energies and forces on each atom were converged to 10^-5^ eV and 0.02 eV/Å, respectively. Spin polarization is turned on for all calculations of models containing Fe elements. We used a Γ-centered *k*-point separation length of 0.04 2*π* Å^−1^ for all structural optimization and 0.03 2*π* Å^−1^ for electronic structure calculations (*e.g.*, Density of states and energy bands). All the above *k*-points and highly symmetric *k*-point paths for calculating energy bands were automatically generated by *VASPKIT*.^[6]^ Charge density and crystal structures were plotted by *VESTA* 3.5.8.^[7]^

The slab structure was constructed from anatase TiO_2_ structure into a 2×1 supercell of 4-layer (101) surface structure which consists of 32 Ti atoms and 64 O atoms, and the lower two layers were fixed during the optimization. A 15 Å vacuum layer was added in the *z*-direction of all the slab structures and dipole correction was considered to avoid interactions with the symmetry switching off. When determining the surface termination of the slab model, the one with the lowest surface energy was selected. In simulating the Fe-doped TiO_2_ slab structure, we referenced experimental results, which showed that with increasing Fe doping, the number of oxygen vacancies in the system also increased. In the pristine-TiO_2_ slab structure, each Fe atom added generates one oxygen vacancy (oxygen vacancy may be located on the surface or subsurface), leading to a lower coordination number of Fe compared to Ti. Based on the Spherical Aberration Transmission Electron Microscope characterization, the optimal-performing TiO_2_-Fe_1_ sample shows Fe dispersed on TiO_2_ nanosheets. Therefore, one Ti catalytic site is replaced with Fe, and the Bridge oxygen atoms from two original Ti catalytic sites are removed to create oxygen vacancies. For the TiO_2_-Fe_2_ sample with a higher doping level, both Ti catalytic sites in the original structure are replaced with Fe, and an oxygen vacancy is created in the subsurface layer.

The energies of small molecules involved in the calculations, such as CO_2_, CH_4_, H_2_O, and H_2_, are obtained by DFT calculations. The Gibbs free energy values for CO and O_2_ are indirectly calculated from the Gibbs free energy changes of reactions as follows, with data sourced from the *NIST-JANAF Thermochemical Tables*.^[8]^

$$CO_{2}\left( g \right)+H_{2}\left( g \right)\to CO\left( g \right)+H_{2}O\left( l \right) \Delta G=0.21 eV$$

$$\Delta G= \Delta_{f}G_{m}^{0}\left( H_{2}O\left( l \right) \right)+\Delta_{f}G_{m}^{0}\left( CO\left( g \right) \right)-\Delta_{f}G_{m}^{0}\left( H_{2}\left( g \right) \right)-\Delta_{f}G_{m}^{0}\left( {CO}_{2}\left( g \right) \right)$$

$$O_{2}\left( g \right)+{2H}_{2}\left( g \right)\to2H_{2}O\left( l \right) \Delta G=-4.92 eV$$

| Gibbs free energy of formation | Value (eV) |
| --- | --- |
| $\Delta_{f}G_{m}^{0}\left( H_{2}O\left( l \right) \right)$ | -2.46 |
| $\Delta_{f}G_{m}^{0}\left( CO\left( g \right) \right)$ | -1.42 |
| $\Delta_{f}G_{m}^{0}\left( H_{2}\left( g \right) \right)$ | 0 |
| $\Delta_{f}G_{m}^{0}\left( {CO}_{2}\left( g \right) \right)$ | -4.09 |

The correction values for the Gibbs free energy ($\Delta G_{correction}$) relative to the gas phase and adsorbed species are calculated using the *VASPKIT* program as the following equations[6, 9].

$$G(T)=E_{DFT}+\Delta G_{correction}$$

$$\Delta G_{correction}= \Delta ZPE+ \int_{0}^{T} C_{p} dT-T\Delta S$$

$$ZPE= \sum_{i} \frac{1}{2hv_{i}}$$

$$\Theta_{i}= \frac{hv_{i}}{k}$$

$$S= \sum_{i} R\left[ -\ln\left( 1-e^{-\frac{\Theta_{i}}{T}} \right)+ {\frac{\Theta_{i}}{T}\left( e^{-\frac{\Theta_{i}}{T}}-1 \right)}^{-1} \right]$$

Where $\Delta ZPE$ is the change in zero-point energy, $C_{p}$ is the heat capacity at constant pressure, $\Delta S$ is the change in entropy, and $\Theta$ is the characteristic temperature of vibration, $h$ is the Planck constant, $k$ is the Boltzmann constant, $v$ is the computed vibrational frequencies.

| Species | *E*_DFT_ | $\Delta G_{correction}$ | $G(T)$ |
| --- | --- | --- | --- |
| H_2_(g) | -6.77 | -0.04 | -6.81 |
| H_2_O(l) | -14.22 | -0.002 | -14.22 |
| O_2_(g) | \ | \ | -9.90 |
| CO_2_(g) | -22.95 | -0.26 | -23.21 |
| CO(g) | \ | \ | -15.59 |
| CH_4_(g) | -24.04 | 0.71 | -23.33 |

The temperature $(T)$ was set to be 298.15 K and the partial pressure of the gas phase species was 1 bar (0.035 bar for H_2_O(l)).

The Gibbs free energies of each elementary reaction in the CO_2_ reduction pathway were calculated by following equations.

$$\Delta G_{1}= G\left( *CO_{2} \right)-G\left( * \right)-G(CO_{2})$$

$$\Delta G_{2}= G\left( *COOH \right)-G\left( CO_{2} \right)-0.5\times G(H_{2})$$

$$\Delta G_{3}= G\left( *CO \right)+G\left( H_{2}O \right)-G\left( *COOH \right)-0.5\times G\left( H_{2} \right)$$

*CO may continue to be protonated to *CHO or desorb to produce CO and *：

$$\Delta G_{4}=G\left( *CHO \right)-G\left( *CO \right)-0.5\times G\left( H_{2} \right)$$

$$\Delta G_{5}=G\left( CO \right)+G\left( * \right)-G\left( *CO \right)$$

$$\Delta G_{6}=G\left( *CH_{2}O \right)-G\left( *CHO \right)-0.5\times G\left( H_{2} \right)$$

$$\Delta G_{7}=G\left( *CH_{3}O \right)-G\left( *CH_{2}O \right)-0.5\times G\left( H_{2} \right)$$

Next if *CH_3_OH intermediate is produced：

$$\Delta G_{8}= G\left( *CH_{3}OH \right)-G\left( *CH_{3}O \right)-0.5\times G(H_{2})$$

$$\Delta G_{9}= G\left( *OH \right)+G\left( CH_{4} \right)-G\left( *CH_{3}OH \right)-0.5\times G\left( H_{2} \right)$$

$$\Delta G_{10}=G\left( * \right)+G\left( H_{2}O \right)-G\left( *OH \right)-0.5\times G(H_{2})$$

if *O intermediate is produced：

$$\Delta G_{8}= G\left( *O \right)+G\left( CH_{4} \right)-G\left( *CH_{3}O \right)-0.5\times G(H_{2})$$

$$\Delta G_{9}= G\left( *OH \right)-G\left( *O \right)-0.5\times G\left( H_{2} \right)$$

$$\Delta G_{10}=G\left( * \right)+G\left( H_{2}O \right)-G\left( *OH \right)-0.5\times G(H_{2})$$

The Gibbs free energy of adsorption *G*_ads_ is defined as following equation:

$$G_{ads}=G_{total}-G_{slab}-G_{species}$$

where *G*_total_, *G*_slab_, and *G*_species_ stand for the Gibbs free energy of adsorption configurations, the energy of slab structure, and the energy of adsorption species, respectively.

**Figures**


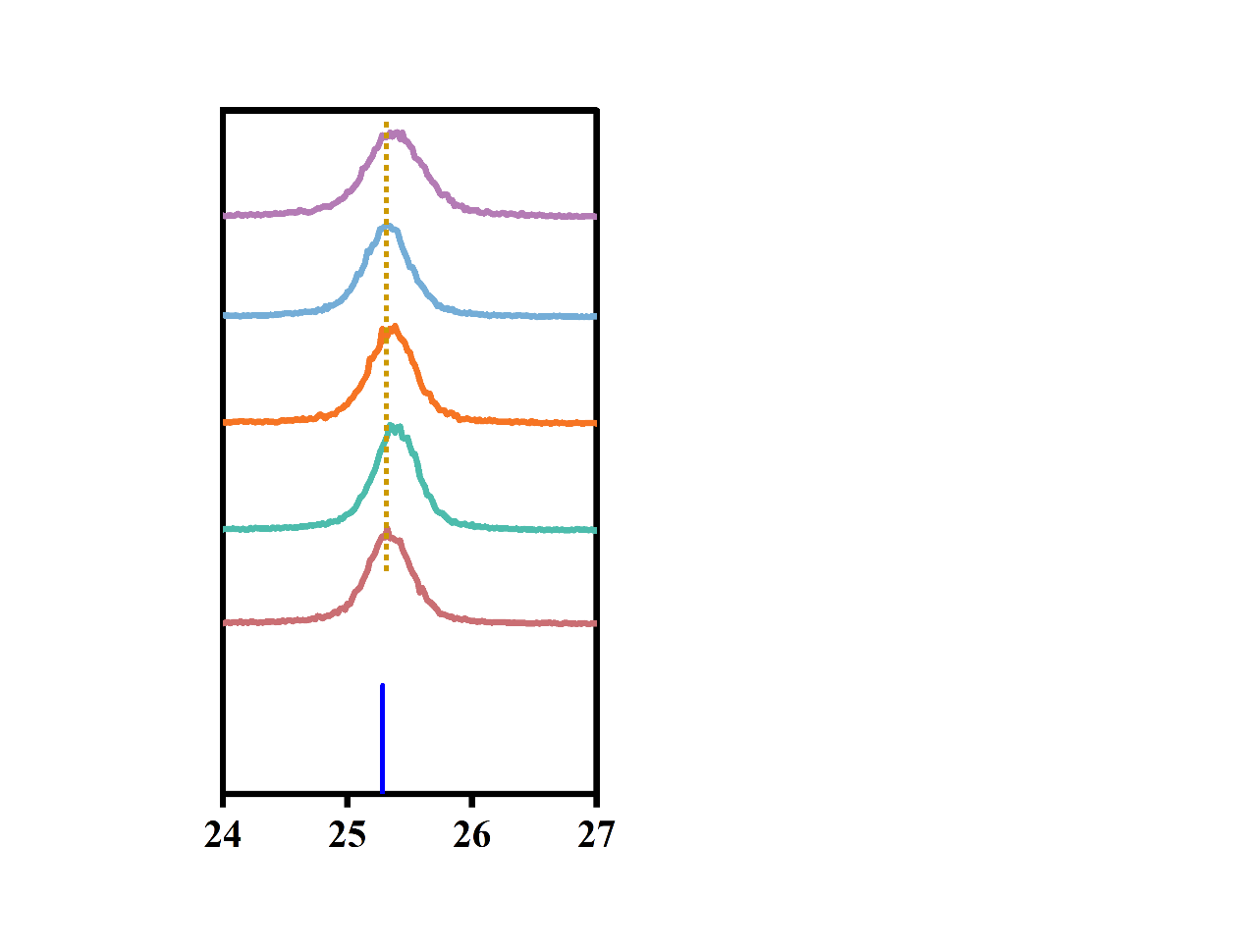


**Figure S1.** Local magnification of XRD diffraction angles 24°-27°.


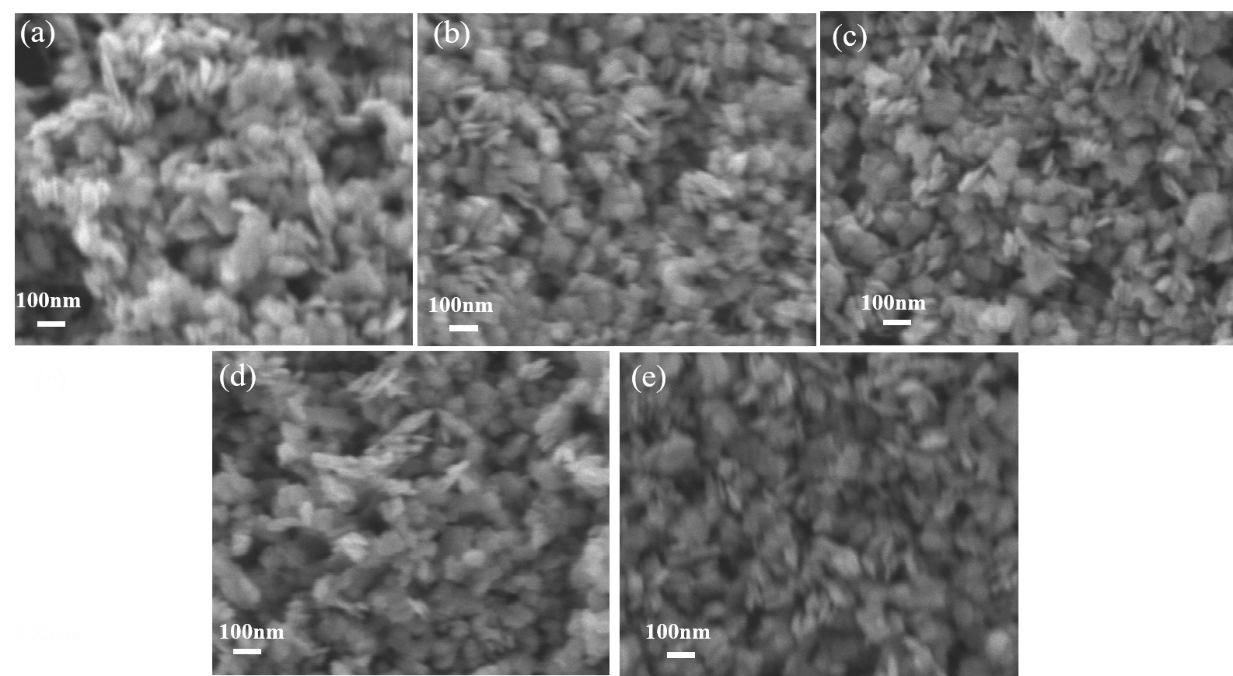
**Figure S2.** SEM images of different materials: (a) TiO_2_. (b) TiO_2_-1Fe. (c) TiO_2_-2Fe. (d) TiO_2_-3Fe.(e) TiO_2_-4Fe.


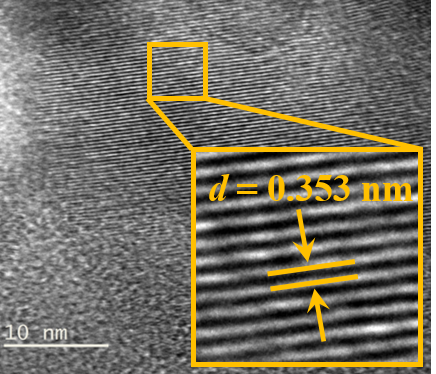


**Figure S3.** HRTEM images of pristine TiO_2_-2Fe ample.


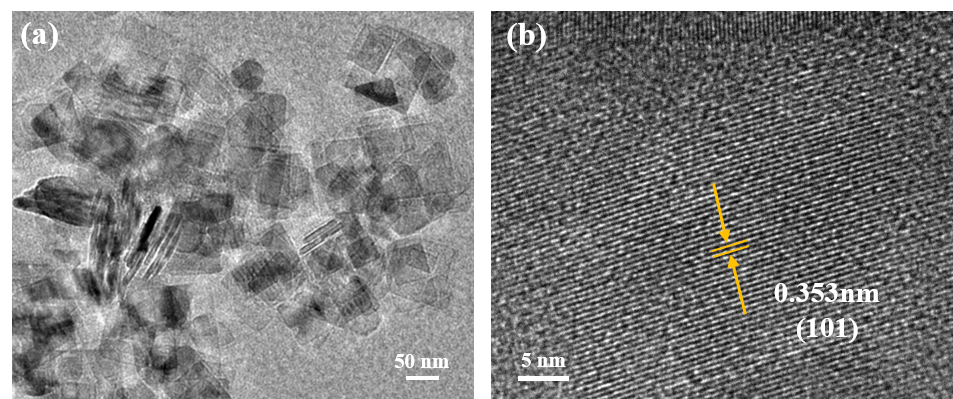
**Figure S4.** a) TEM images of pristine TiO_2_. b) HRTEM images of pristine TiO_2_.


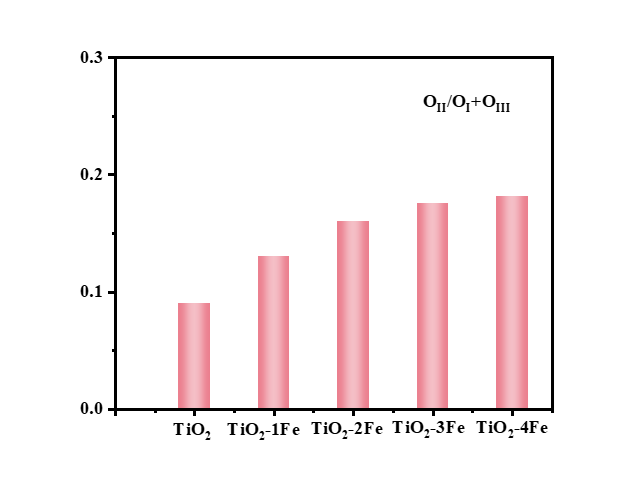


**Figure S5.** Quantitative analysis of oxygen in XPS according to peak area.


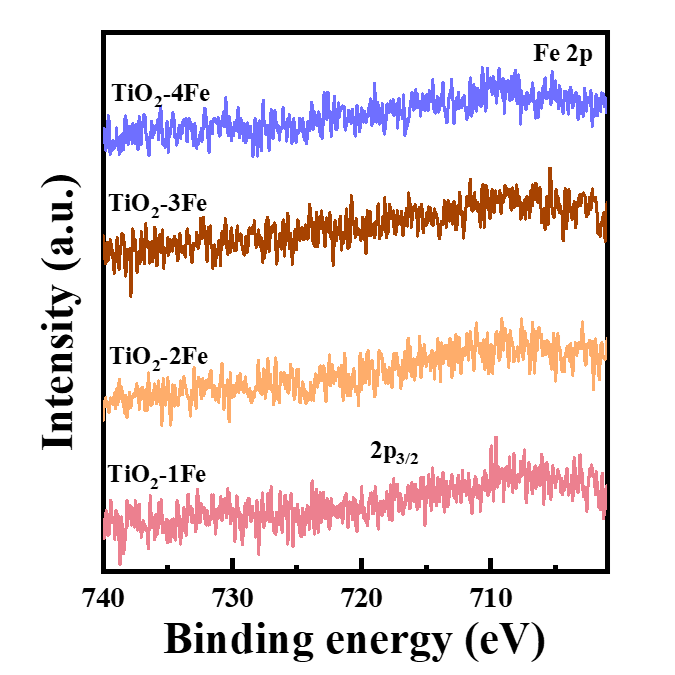


**Figure S6.** XPS spectra of Fe 2p for TiO_2_-Fe samples.


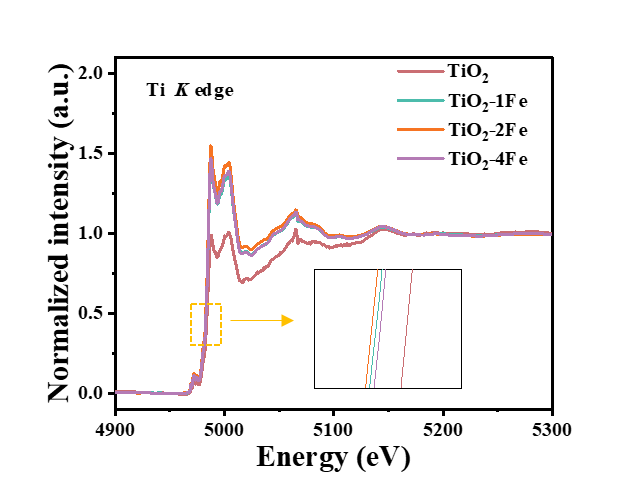


**Figure S7.** Ti *K*-edge XANES spectra.


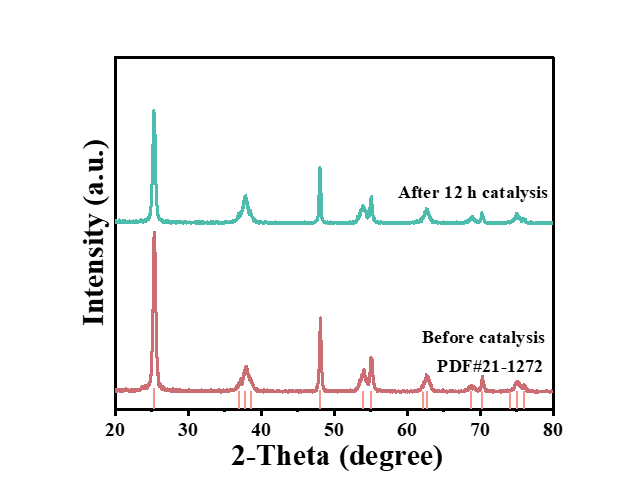


**Figure S8.** XRD pattern of TiO_2_-2Fe before and after 12 hours catalysis reaction.


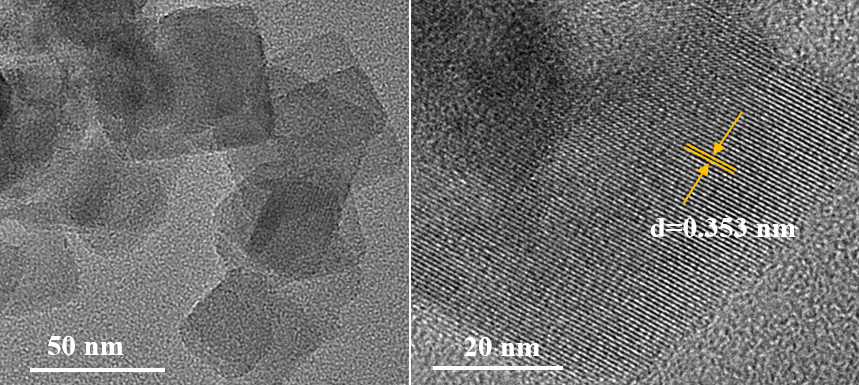


**Figure S9.** TEM of TiO_2_-2Fe sample after 12 hours catalysis reaction.


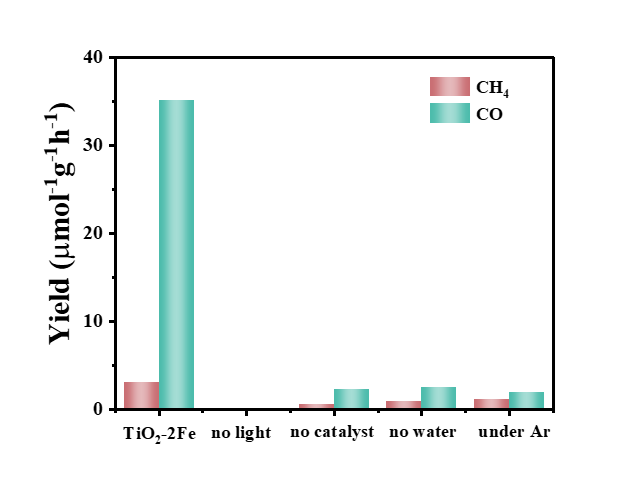


**Figure S10.** Control experiments under no light, no catalyst, no water, Ar atmosphere.


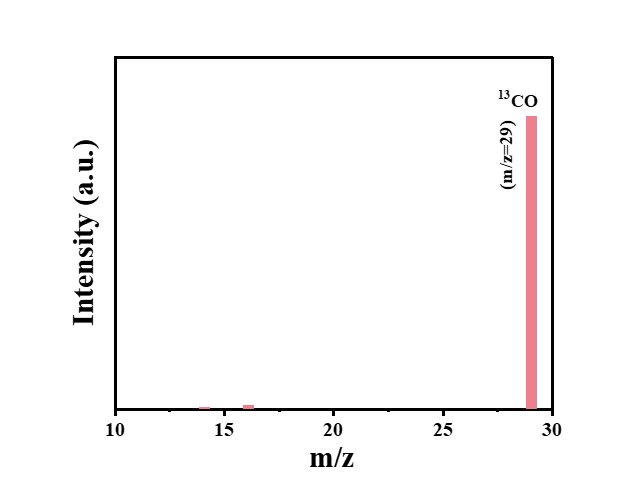


**Figure S11.** GC-MS analysis for ^13^CO_2_ labeling experiment of TiO_2_-2Fe.


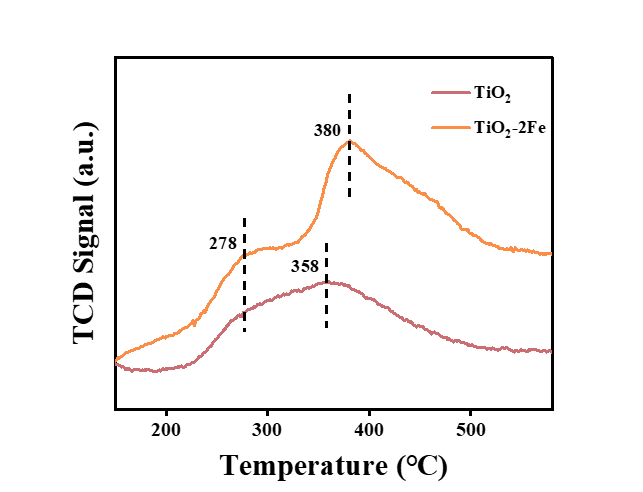


**Figure S12.** CO_2_-TPD curves for TiO_2_ and TiO_2_-2Fe.


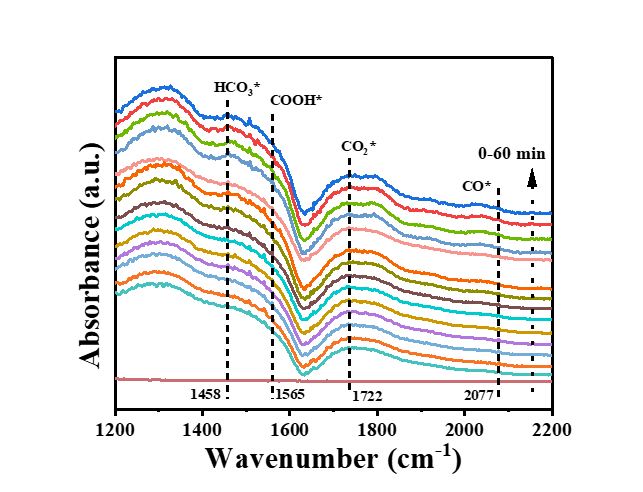


**Figure S13.** In-*situ* FTIR spectroscopy characterization for TiO_2_.


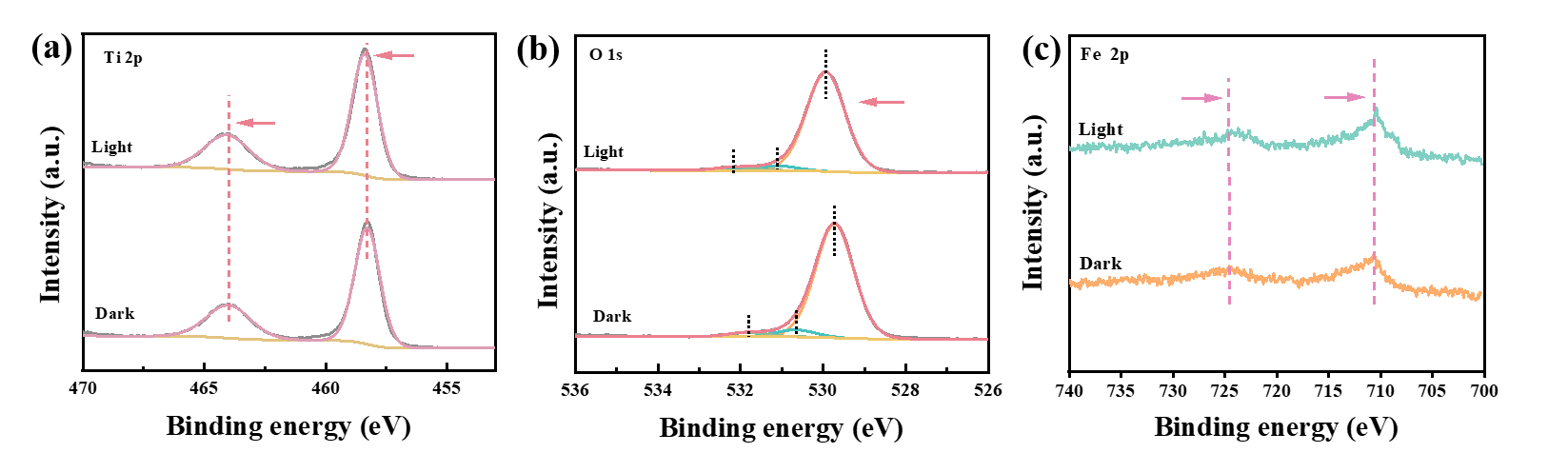


**Figure S14.** In-situ XPS spectra of TiO_2_-Fe sample: a) Ti 2p, b) O 1s and c) Fe 2p under light irradiation or in the dark.


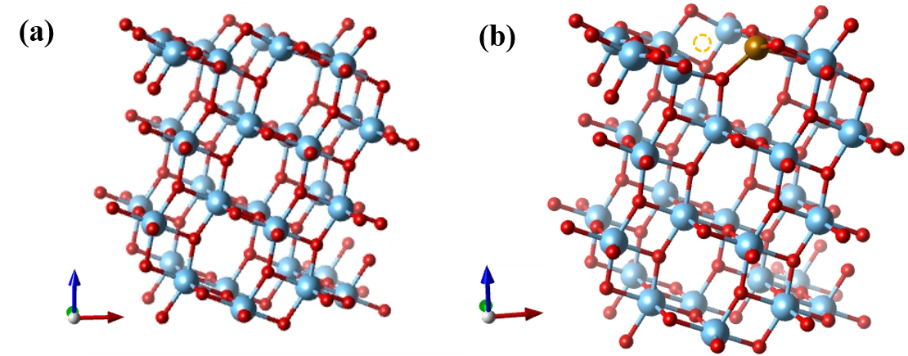


**Figure S15.** Crystal structure models of a) TiO_2_ and b) TiO_2_-Fe_1_. The red, blue, yellow spheres and orange circle represent the O atom, the Ti atom, the Fe atom, and O vacancy, respectively.


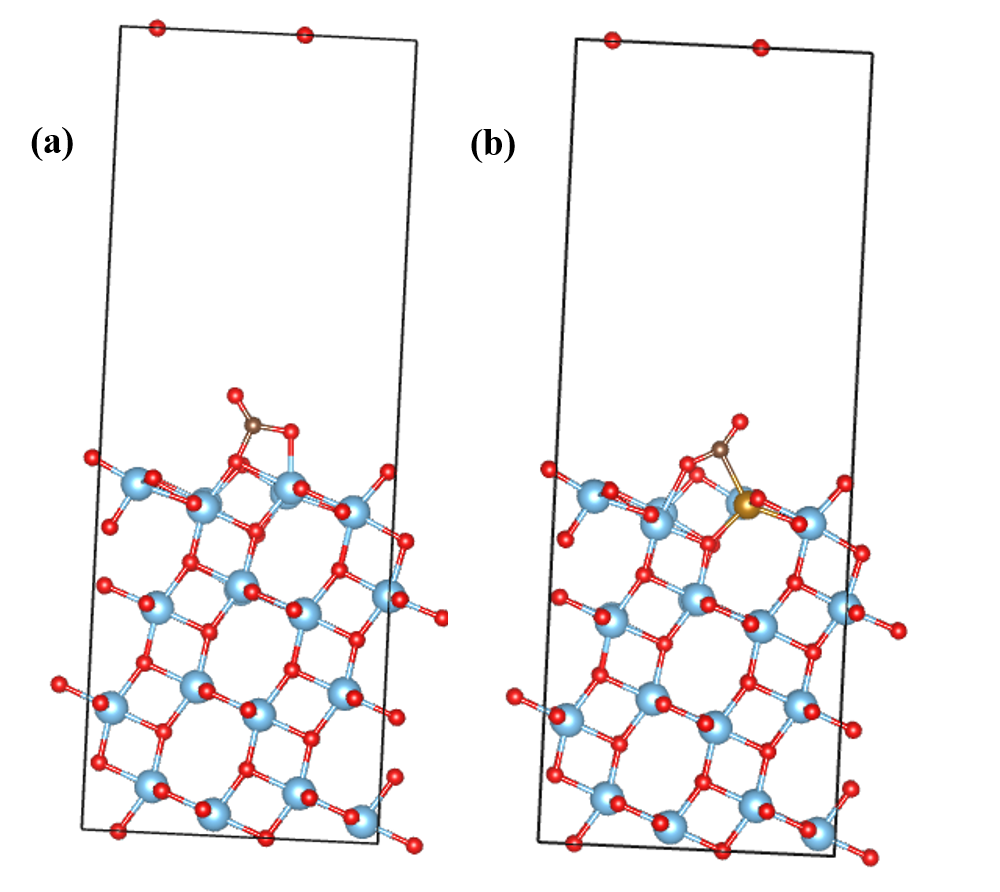


**Figure S16.** The different structure with CO_2_ adsorbed on the surface. The red, blue, yellow, and brown spheres represent the O atom, the Ti atom, the Fe atom, and the C atom, respectively. a) TiO_2_ and b) TiO_2_-Fe_1_.


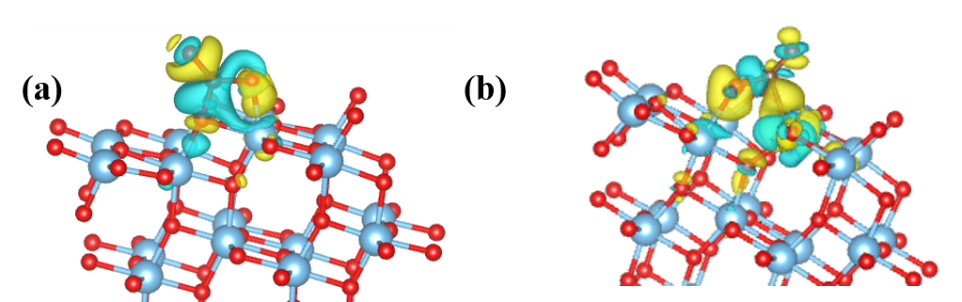


Δ*G*_ads_ (CO_2_) =0.5297 eV Δ*G*_ads_ (CO_2_) =0.3436 eV

Δ*q*= 3.33 e^-^ Δ*q*= 3.64 e^-^

**Figure S17.** Charge density difference distribution of optimized CO_2_ adsorbed on TiO_2_ a), CO_2_ adsorbed on TiO_2_-Fe_1_ b). Light blue and yellow represent charge loss and charge accumulation, respectively. The red, blue, yellow, and brown spheres represent the O atom, the Ti atom, the Fe atom, and the C atom, respectively.


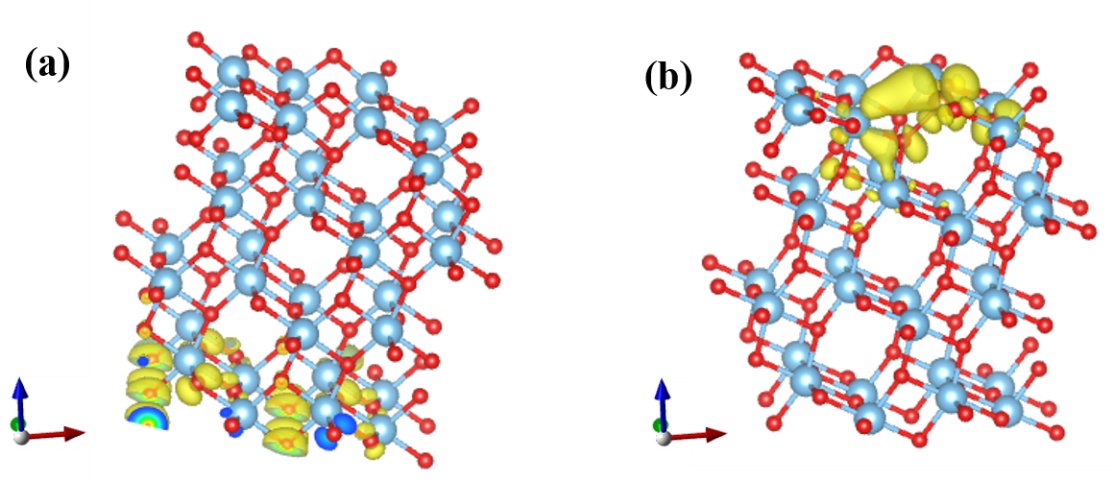


*G*_ads_ (CO) =-0.13 eV Δ*G*_ads_ (CO) =1.99 eV

**Figure S18.** CO adsorbed the molecular charge densities for VBM of (a) TiO_2_ and (b) TiO_2_-Fe_1_. Light blue and yellow represent charge loss and charge accumulation, respectively. The red, blue, yellow, and brown spheres represent the O atom, the Ti atom, the Fe atom, and the C atom, respectively.


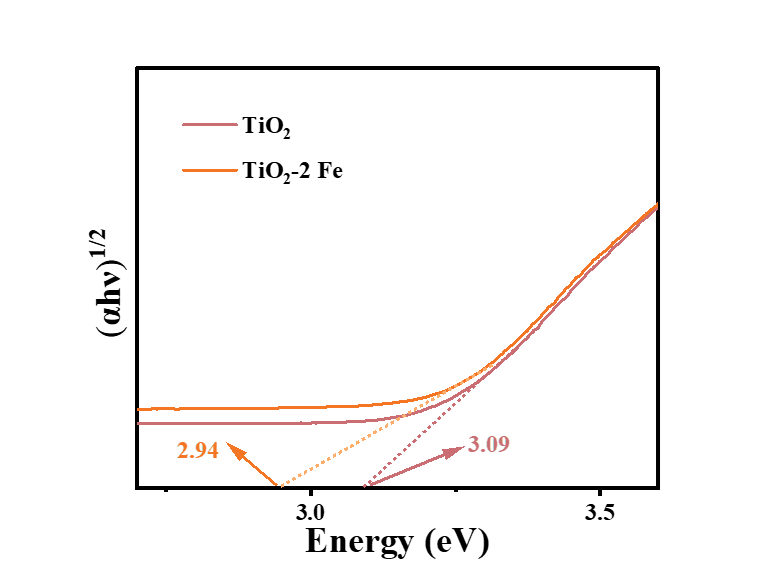


**Figure S19.** The band gap energy of the TiO_2_ and TiO_2_-2Fe.


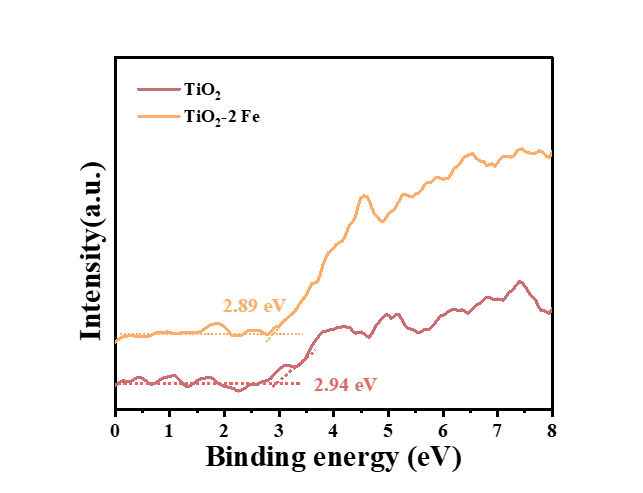


**Figure S20.** Valence band structure of the TiO_2_ and TiO_2_-2Fe.


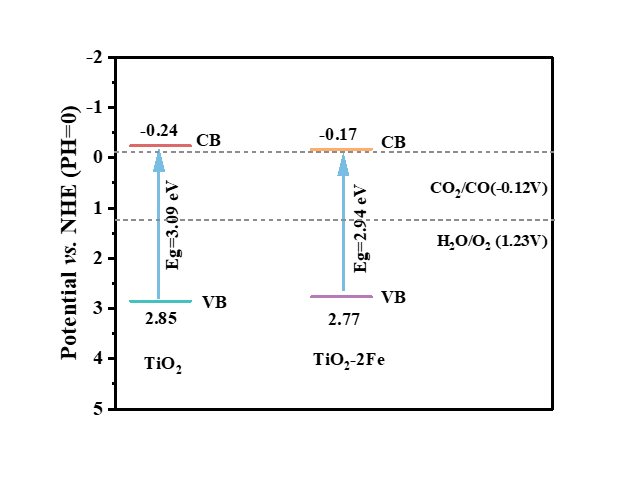


**Figure S21.** The schematic of energy band alignments for TiO_2_ and TiO_2_-2Fe.


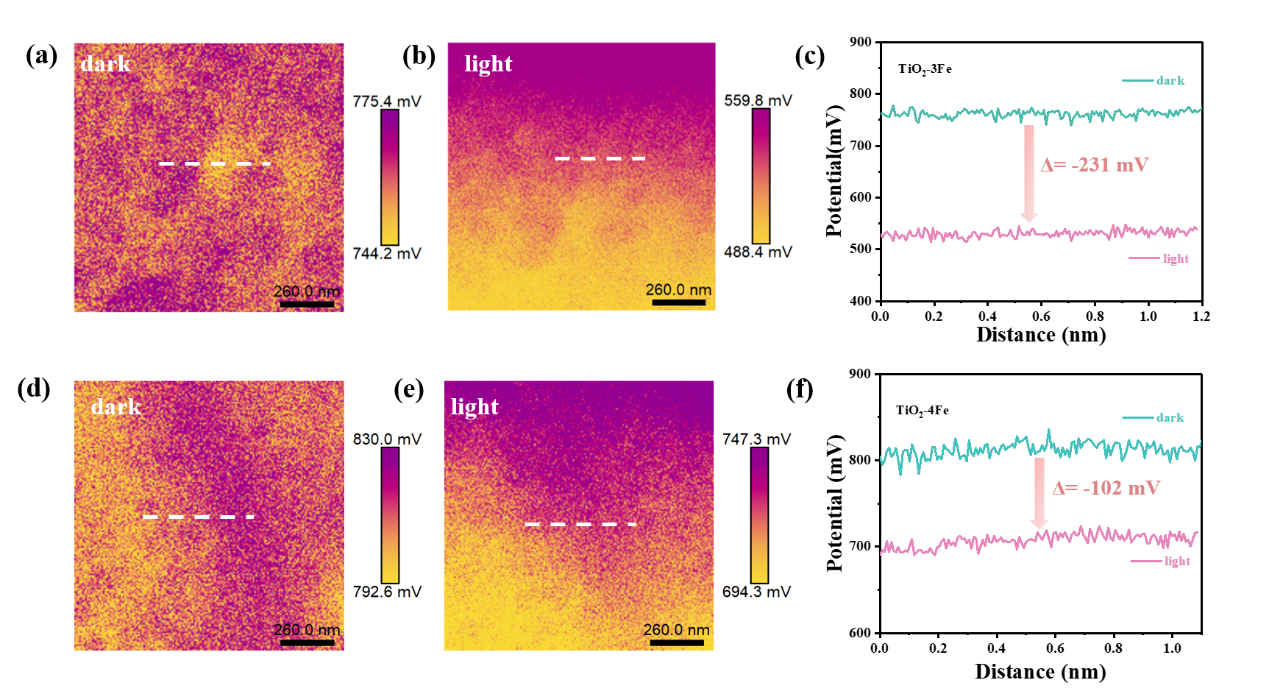


**Figure S22.** a-f) KPFM pictures of TiO_2_-3Fe and TiO_2_-4Fe. a, d) corresponding the surface potential distribution under darkness and b, e) under light. c, f) The line-scanning surface potential of TiO_2_-3Fe and TiO_2_-4Fe, respectively.


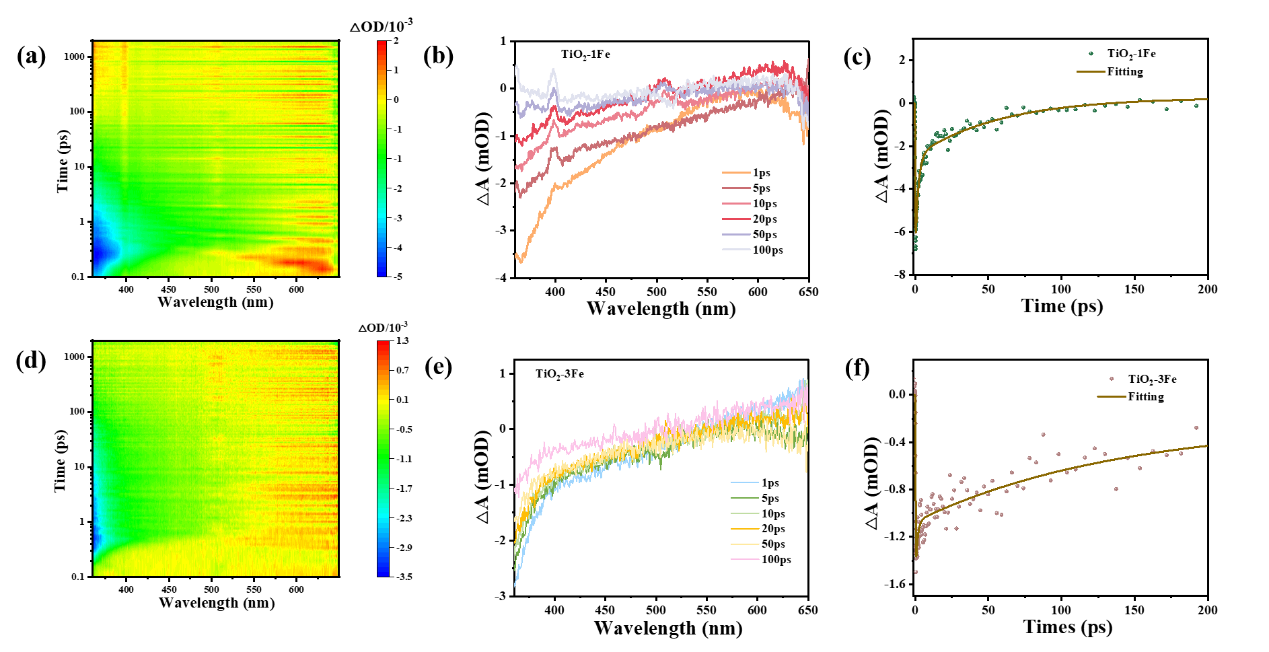


**Figure S23.** a-f) fs-TAS monitors the photogenerated carrier dynamics for TiO_2_-1Fe and TiO_2_-3Fe. a, d) 2D pseudo color pictures. b, e) Transient fs-TA spectra under different probe delays. c, f) fs-TAS kinetics decay profile probed at 380 nm.


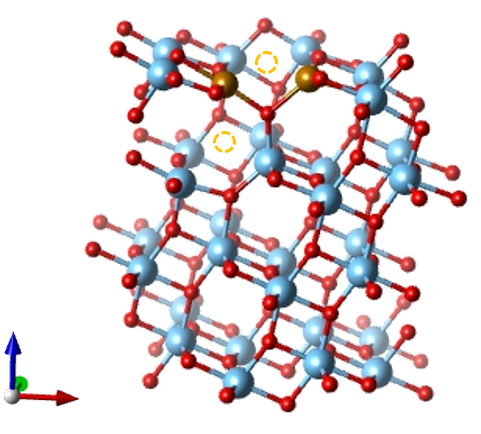


**Figure S24.** Crystal structure models of TiO_2_-Fe_2_. The red, blue, yellow spheres and orange circle represent the O atom, the Ti atom, the Fe atom, and O vacancy, respectively.


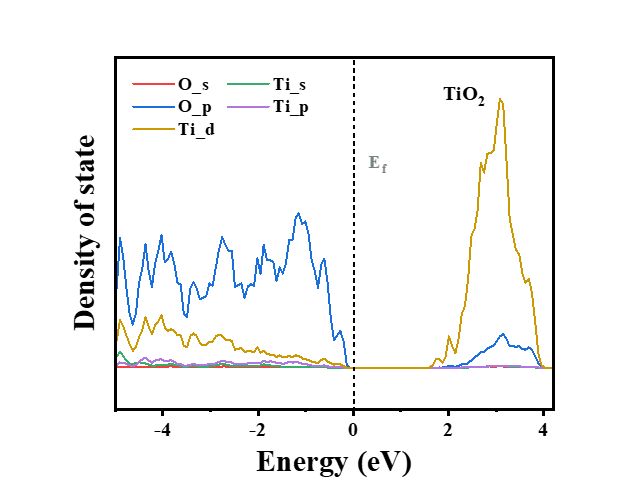


**Figure S25.** The PDOS for Ti and O elements of TiO_2_.


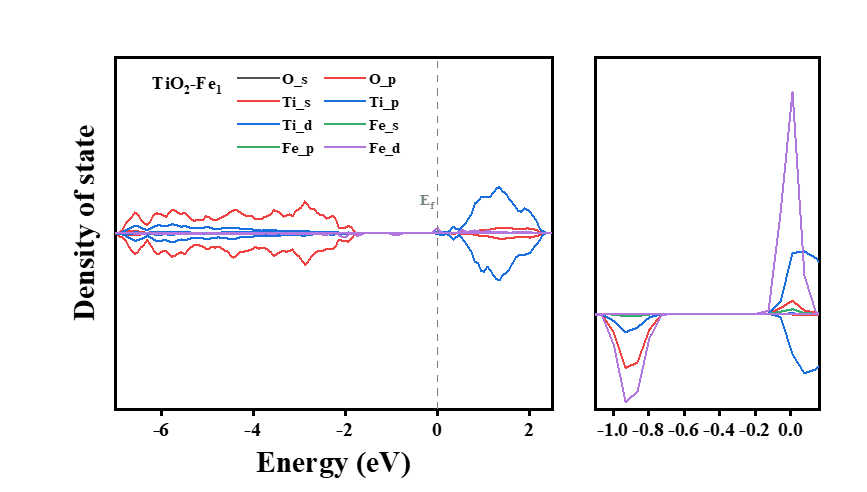


**Figure S26.** The PDOS for Ti and O elements of TiO_2_-Fe_1_.


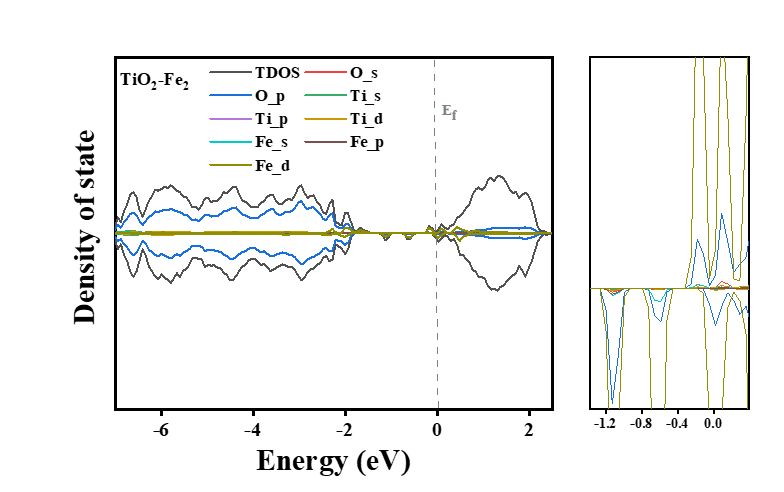


**Figure S27.** The enlarged PDOS for Ti and O elements of TiO_2_-Fe_2_.

**Table S1.** The mass fraction of Fe of TiO_2_-Fe samples were calculated by IMPE

| Sample | Fe (wt%) |
| --- | --- |
| TiO_2_-1 Fe | 0.147 |
| TiO_2_-2 Fe | 0.213 |
| TiO_2_-3 Fe | 0.377 |
| TiO_2_-4 Fe | 0.965 |

**Table S2.** The contents of lattice oxygen, O_v_ and surface adsorbed oxygen for the sample from XPS spectra analysis

| Sample | Ti-O/% | O_v_/% | S_ur_-OH/% |
| --- | --- | --- | --- |
| TiO_2_ | 86.34 | 8.4 | 5.26 |
| TiO_2_-1 Fe | 82.71 | 11.51 | 5.78 |
| TiO_2_-2 Fe | 79.87 | 14.2 | 5.92 |
| TiO_2_-3 Fe | 79.22 | 14.9 | 5.88 |
| TiO_2_-4 Fe | 78.35 | 15.35 | 6.3 |

**Table S3.** The specific yield rate (μmol g^-1^) of CO and CH_4_ for TiO_2_-2Fe

| Time  (2h) | First cycle  CH_4_ CO | Second cycle  CH_4_ CO | Third cycle  CH_4_ CO | Fourth cycle  CH_4_ CO | Fifth cycle  CH_4_  CO |
| --- | --- | --- | --- | --- | --- |
|  | 7.0574 71.1874 | 5.8612 68.866 | 6.2496 54.6402 | 4.3076 44.7024 | 4.3076 44.7024 |
| decay |  | 16.94% 4.22% | 11.44% 23.24% | 38.96% 37.29% | 38.96% 37.29% |

**Table S4.** Performance comparison of catalysts for photocatalytic CO_2_ reduction to CO

| Catalyst | Rection condition | | CO yield (μmol g^-1^ h^-1^) | | Reference |
| --- | --- | --- | --- | --- | --- |
| TiO_2_-2Fe | Pure CO_2_ gas, Gas-solid | 35.12 | | | **This work** |
| TiO_2_-Cu | Pure CO_2_ gas, Gas-solid | 15.27 | | | S10 |
| TiO_2_/CsPbBr_3_ | Pure CO_2_ gas, Liquid-solid, triethanolamine, CoCl_2_ and 2, 2-bipyridine as cocatalyst | 12.5 | | | S11 |
| CTU/TiO_2_ | Pure CO_2_ gas (0.1Mpa), Gas-solid | 31.32 | | | S12 |
| Ni/TiO_2_ | Pure CO_2_ gas, Gas-solid | 22.654 | | | S13 |
| Ag/TiO_2_ | Pure CO_2_ gas, Liquid-solid, | 5.2 | | | S14 |
|  | 0.1M NaHCO_3_ |  | | |  |
| TiO_2_-101 | Pure CO_2_ gas, Gas-solid | 8.3 | | | S15 |
| 2.5 wt%  Cu/Cu^+^@TiO_2_ | Gas phase, 0.1 M Na_2_SO_3_ as the sacrificial agent | 1.7 | | S16 | |
| Pt/TiO_2_  photonic crystals | Gas phase, 320-780 nm | 0.2 | | S17 | |
| TB-H_2_O_2_ | Pure CO_2_ gas, Liquid-solid | 29.1 | | S18 | |

**Table S5.** Fluorescence lifetime fitting data of TiO_2_ and TiO_2_-Fe catalyst

| Sample | *τ*_1_ (ns) | B_1_(%) | *τ*_2_ (ns) | B_2_(%) | *τ*_3_ (ns) | B_3_(%) | *τ*_ave_(ns) |
| --- | --- | --- | --- | --- | --- | --- | --- |
| TiO_2_ | 0.654 | 30.24 | 3.419 | 36.61 | 42.24 | 33.15 | 15.453 |
| TiO_2_-1 Fe | 0.388 | 30.30 | 2.644 | 25.07 | 44.74 | 44.64 | 20.752 |
| TiO_2_-2 Fe | 0.6821 | 14.49 | 3.734 | 19.68 | 47.69 | 65.82 | 32.223 |
| TiO_2_-3 Fe | 0.498 | 20.53 | 3.576 | 28.18 | 44.97 | 51.29 | 24.175 |
| TiO_2_-4 Fe | 0.381 | 34.05 | 2.767 | 27.73 | 43.65 | 38.22 | 17.5303 |

**Table S6.** Kinetic parameters of the attenuation process fitted to the sample at 380 nm

| Sample | *τ*_1_ (ps) | *τ*_2_ (ps) |
| --- | --- | --- |
| TiO_2_ | 1.95 | 52.7 |
| TiO_2_-1 Fe | 2.02 | 57.93 |
| TiO_2_-2 Fe | 4.59 | 139 |
| TiO_2_-3 Fe | 1.34 | 111.4 |

**References**

[1] G. Kresse, J. Furthmüller, *Phys. Rev. B* **1996**, *54*, 11169-11186.

[2] G. Kresse, J. Furthmüller, *Comput. Mater. Sci.* **1996,** *6*, 15-50.

[3] P.E. Blöchl, *Phys. Rev. B* **1994,** *50*, 17953-17979.

[4] J.P. Perdew, K. Burke, M. *Phys. Rev. Lett.* **1996,** *77*, 3865-3868.

[5] S. Grimme, J. Antony, S. Ehrlich, H. Krieg, *J. Chem. Phys.* **2010**, *132*, 154104.

[6] V. Wang, N. Xu, J.-C. Liu, G. Tang, W.-T. Geng, *Comput. Phys. Commun.* **2021,** *267*, 108033.

[7] K. Momma, F. Izumi, *J. Appl. Crystallogr.* **2011**, *44*, 1272-1276.

[8] M.W. Chase, Jr., J.L. Curnutt, J.R. Downey, Jr., R.A. McDonald, A.N. Syverud, E.A. Valenzuela, *J. Phys. Chem. Ref. Data* **1982**, *11*, 695-940.

[9] B. Lu, C. Wahl, R. dos Reis, J. Edgington, X.K. Lu, R. Li, M.E. Sweers, B. Ruggiero, G.T.K.K. Gunasooriya, V. Dravid, L.C. Seitz, *Nat. Catal.* **2024**, *7*, 868-877.

[10] K. N. Zhu, Q. Zhu, M. P. Jiang, Y. W. Zhang, Z. Y. Shao, Z. B. Geng, X. Y. Wang, H. Zeng, X. F. Wu, W. Zhang, K. K. Zhang, S. H. Feng, *Angew. Chem. Int. Ed.* **2022**, *61*, e202207600.

[11] L. X. Wang, J. Y. Qiu, N. Wu, X. Q. An, *J. Colloid Interface Sci.* **2023**, *629*, 206-214.

[12] L. Wang, P. X. Jin, S. H. Duan, H. D. She, J. W. Huang, Q. Z. Wang, *Sci. Bull.* **2019**, *64*, 926-933.

[13] Z. H. Li, W. C. Bai, D. Liu, B. C. Han, Y. M. Liang, J. Qi, *Sep. Purif. Technol.* **2024**, *330*,125250.

[14] L. Jin, E. Shaaban, S. Bamonte, D. Cintron, S. Shuster, L. Zhang, G. H. Li, J. He, *ACS Appl. Mater. Interfaces* **2021**, *13*, 38595-38603.

[15] J. Y. Wang, Z. Xiong, Y. C. Zhao, J. Y. Zhang, *ACS Appl. Mater. Interfaces* **2024**, *16*, 67743-67751.

[16] S. Zhu, X. F. Chen, Z. C. Li, X. Y. Ye, Y. Liu, Y. Chen, L. Yang, M. Chen, D. Q. Zhang, G. S. Li, *Appl. Catal. B* **2020**, *264*, 118515.

[17] J. Jiao, Y. Wei, K. Chi, Z. Zhao, A. Duan, J. Liu, G. Jiang, Y. Wang, X. Wang, C. Han, P. Zheng, *Energy Technol.* **2017**, 5, 877-883.

[18] C. C. Jia, B. J. Wan, W. G. Liu, L. G. Qi, X. X. Liu, X. X. Han, A. L. Gao, J. Liu,

*Adv. Funct. Mater.* **2024**, *34*, 2311663.
